# Fis1 phosphorylation by Met promotes mitochondrial fission and hepatocellular carcinoma metastasis

**DOI:** 10.1038/s41392-021-00790-2

**Published:** 2021-12-01

**Authors:** Yan Yu, Xiao-Dan Peng, Xiao-Jun Qian, Kai-Ming Zhang, Xiang Huang, Yu-Hong Chen, Yun-Tian Li, Gong-Kan Feng, Hai-Liang Zhang, Xue-Lian Xu, Shun Li, Xuan Li, Jia Mai, Zhi-Ling Li, Yun Huang, Dong Yang, Li-Huan Zhou, Zhuo-Yan Zhong, Jun-Dong Li, Rong Deng, Xiao-Feng Zhu

**Affiliations:** 1 State Key Laboratory of Oncology in South China, Collaborative Innovation Center for Cancer Medicine, Guangdong Key Laboratory of Nasopharyngeal Carcinoma Diagnosis and Therapy, Sun Yat-Sen University Cancer Center, Guangzhou, 510060 China; 2 The First Affiliated Hospital of USTC, Division of Life Sciences and Medicine, University of Science and Technology of China, Hefei, Anhui 230001 P.R. China; 3 Department of Medical Bioinformatics, Zhongshan School of Medicine, Sun Yat-Sen University, Guangzhou, 510080 China; 4 The First Affiliated Hospital of Xinxiang Medical College, Weihui, Henan 453100 China; 5 Department of Gynecological Oncology, Sun Yat-Sen University Cancer Center, Guangzhou, 510060 China

**Keywords:** Oncogenes, Metastasis

## Abstract

Met tyrosine kinase, a receptor for a hepatocyte growth factor (HGF), plays a critical role in tumor growth, metastasis, and drug resistance. Mitochondria are highly dynamic and undergo fission and fusion to maintain a functional mitochondrial network. Dysregulated mitochondrial dynamics are responsible for the progression and metastasis of many cancers. Here, using structured illumination microscopy (SIM) and high spatial and temporal resolution live cell imaging, we identified mitochondrial trafficking of receptor tyrosine kinase Met. The contacts between activated Met kinase and mitochondria formed dramatically, and an intact HGF/Met axis was necessary for dysregulated mitochondrial fission and cancer cell movements. Mechanically, we found that Met directly phosphorylated outer mitochondrial membrane protein Fis1 at Tyr38 (Fis1 pY38). Fis1 pY38 promoted mitochondrial fission by recruiting the mitochondrial fission GTPase dynamin-related protein-1 (Drp1) to mitochondria. Fragmented mitochondria fueled actin filament remodeling and lamellipodia or invadopodia formation to facilitate cell metastasis in hepatocellular carcinoma (HCC) cells both in vitro and in vivo. These findings reveal a novel and noncanonical pathway of Met receptor tyrosine kinase in the regulation of mitochondrial activities, which may provide a therapeutic target for metastatic HCC.

## Introduction

Liver cancer is the second leading cause of cancer death worldwide because of the high rate of metastasis.^1^ High hepatocyte growth factor (HGF) levels in serum or overexpression of Met in hepatocellular carcinoma (HCC) are closely associated with early recurrence,^2^ and patients with high expression levels of Met usually have low 5-year survival rates after curative surgical resection.^2–5^ Upon activation, the tyrosine residues Y1234 and Y1235 in the kinase domain of Met are phosphorylated, which leads to auto-phosphorylation of the C-terminal multi-substrate docking site, Y1349, and Y1356. Various cytoplasmic effectors, including PI3K, Ras, PLC-γ, Shc, and SHP2, are recruited to the docking site and subsequently activated. Currently, at least 17 Met inhibitors, including JNJ-38877605, GEN-203, and ARQ-197, are undergoing clinical trials.^6^ However, no selective Met inhibitors, such as SU11274 and Tepotinib, have been proven despite the encouraging wave of recent drug approvals for HCC.^7–10^ Due to adverse side reactions and limited therapeutic effects, many clinical trials of selected Met inhibitors were hindered at Stage II or III,^11,12^ There still remains a need for a clearer understanding of the HGF/Met pathway to accelerate Met-targeting strategy development.

The internalization of membrane docking Met after HGF stimulation and activation has been studied for nearly two decades.^13,14^ Endosome-carried Met was supposed to be degraded along with the other contents of mature endosomes fusing with lysosomes.^15,16^ Following endocytosis, Met was found to signal either from the peripheral endosome to fully activate ERK1/2^14^ and Rac1 or from the perinuclear endosome to activate STAT3.^13^ Recently, Met was reported to be located in the cell nucleus and phosphorylate PARP to facilitate BRCA inhibitor resistance.^17^ These findings suggest that Met may be redistributed to multiple organelles after internalization. Kang et al. reported that 38% of proteins whose expression was perturbed by Met inhibition were mitochondrial proteins,^18^ and a selective mitochondrial-targeting Met kinase inhibitor potently killed erlotinib-resistant lung cancer cells,^19^ suggesting that Met-regulated mitochondrial activity is strongly associated with cancer cell survival.

Mitochondria are highly dynamic organelles that are continually undergoing fission and fusion.^20^ Altered mitochondrial dynamics have been linked to abnormal cell functions^21,22^ and many human diseases,^23–26^ including cancers.^26,27^ Studies have shown that excess mitochondrial fission^28,29^ and upregulation of the fission component Fis1-Drp1 are frequently involved in tumorigenesis and metastasis.^28,30–32^ How mitochondrial fission regulates cancer cell movements remains elusive. A few lines of evidence showed that the following fission, fragmented mitochondria were distributed to the leading edge of cancer cells to facilitate the formation of lamellipodia or invadopodia, which is a key step in cell migration and invasion.^33–35^

In the present study, using structured illumination microscopy (SIM) and high spatial and temporal resolution live cell imaging, we found that Met localized at mitochondrial dividing sites and catalyzed Fis1 Tyr38 phosphorylation to promote mitochondrial fission. Fragmented mitochondria thus facilitated the redistribution of mitochondria to the leading edge of HCC cells to fuel actin filament remodeling and lamellipodia or invadopodia formation. We defined a vital role of Met in the regulation of mitochondrial fission to promote metastasis of HCC cells in vitro and in vivo. Our data suggested a novel and noncanonical pathway of Met receptor tyrosine kinase in the regulation of mitochondrial activities, which may provide a therapeutic target for metastatic HCC.

## Results

### HGF stimulates the mitochondrial localization of met

**Fig. 1:**
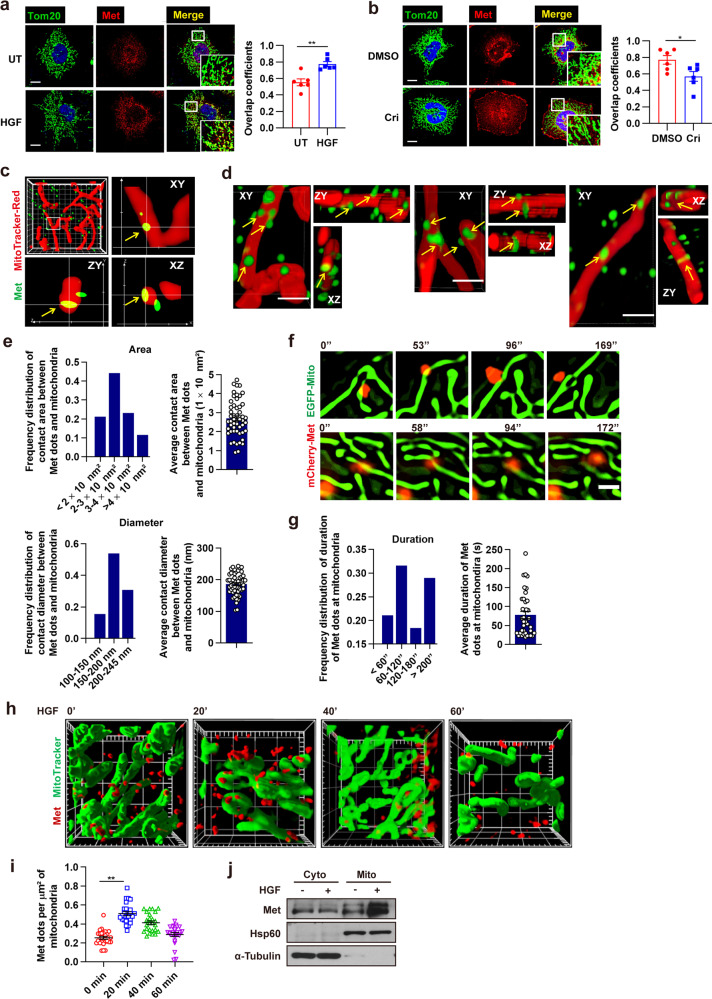
HGF stimulates mitochondrial location of Met. **a**, **b** Huh7 cells were treated with HGF (100 ng/ml, 20 min) (**a**) or crizotinib (1 µM, 1 h) (**b**) and immunofluorescent stained for Met and Tom20, a mitochondrial marker. Images were captured by a confocal microscope. The zoomed images show Met colocalization with mitochondria. Scale bars, 100 µm. Histogram reporting Mander’s overlap coefficients relative to Met colocalization with mitochondria. Error bars represent means ± SEM (*n* = 6 cells, *p < 0.05, **p < 0.01; Student’s *t* test). **c**, **d** Representative 3D SIM images of mitochondrial localization of Met (yellow arrows) in Huh7 cells immunofluorescent stained for Met and mitochondria, showing the cross-section profiles (**c**) and three fields of surface profiles (**d**). Images in (**c**) were captured in *Z*-stacks showing contacts extending more than 200 nm in the *Z*-plane (frame, 10 µm × 10 µm; main calibration, 1 µm). Scale bars of (**d**), 1 µm. **e** Quantification of area (upper) and diameter (lower) of the contacting region between Met dots and mitochondria according to (**c**) and (**d**). Error bars represent means ± SEM (*n* = 52 contacts). **f** Time-lapse SIM live-cell imaging of mitochondrial localization of Met in HeLa cells stably expressing mCherry–Met and EGFP–Mito. Two fields were captured. Scale bars, 1 µm. **g** Quantification of contact duration of Met and mitochondria according to (**f**). Error bars represent means ± SEM (*n* = 38 contacts). **h** Representative 3D SIM images of mitochondrial localization of Met in Huh7 cells treated with HGF (100 ng/ml) for the indicated time. Cells were immunofluorescent stained for Met and mitochondria (frame, 10 µm × 10 µm; main calibration, 1 µm). **i** Quantification of Met docking at mitochondria at indicated time points according to (**h**). Error bars represent means ± SEM (*n* = 25 fields, **p < 0.01; Student’s *t* test). **j** Immunoblot analysis of Met in mitochondrial and cytosolic fractions of Huh7 cells after treated with HGF (100 ng/ml, 20 min). Mitochondria isolation markers, α-Tubulin (cytoplasmic) and Hsp60 (mitochondria) were used as controls

Met kinase has been reported to localize at endosomes, lysosomes, and nucleus,^36,37^ and it forces us to think about the possibility of Met localization at mitochondria. So far, none has been reported about the mitochondrial localization signal (MLS) of Met. To predict the probability of Met import to mitochondria, we analyzed the Met protein sequence by using the MitoProt database.^38^ The MitoProt software predicted MLS and a cleavage site after the first 22 amino acids at the N terminus with a probability score of 0.07. We further identified the localization of Met at mitochondria using conventional confocal microscopy as well as structured illumination super-resolution microscope (SIM). We performed confocal microscopy analysis in Huh7 cells expressing high levels of Met and found that Met had a propensity to colocalize with mitochondria in the absence of any stimuli (Fig. 1a, b). 3D SIM analysis confirmed such colocalization. We observed that Met and mitochondria formed contacts with an average area of 2.70 ± 0.38, 1 × 10^4^ nm^2^, and a diameter of 178.45 ± 11.62 nm in Huh7 cells under 3D SIM (Fig. 1c–e). To observe Met mitochondrial localization dynamically, we subjected HeLa cells that stably expressed Met tagged with mCherry fluorescent protein (mCherry–Met) and mitochondria-targeting enhanced green fluorescent protein (EGFP–Mito) to live SIM. We observed that mCherry–Met translocated into mitochondria and its localization at mitochondria remained for a long period, lasting for ~147.50 ± 9.40 s (Fig. 1f, g and Supplementary Movie S1).

To examine whether Met kinase activity influenced its mitochondrial localization, we employed exogenous recombinant human HGF and Met kinase inhibitor crizotinib in Huh7 cells. Confocal microscopy analysis showed that the overlap between Met and mitochondria was increased upon HGF treatment but reduced with crizotinib treatment (Fig. 1a, b). Under live 3D SIM, we observed that Met and mitochondria formed much more contacts upon HGF treatment, which peaked at 20 min and then fell to baseline afterward, in line with the time feature of Met activation by HGF (Fig. 1h, i). Consistently, subcellular fractionation studies revealed that HGF treatment stimulated Met localization in the mitochondrial fraction (Fig. 1j). Overall, these results show that Met localizes to mitochondria upon HGF stimulation.

### Kinase activity is required for mitochondrial trafficking of met

**Fig. 2:**
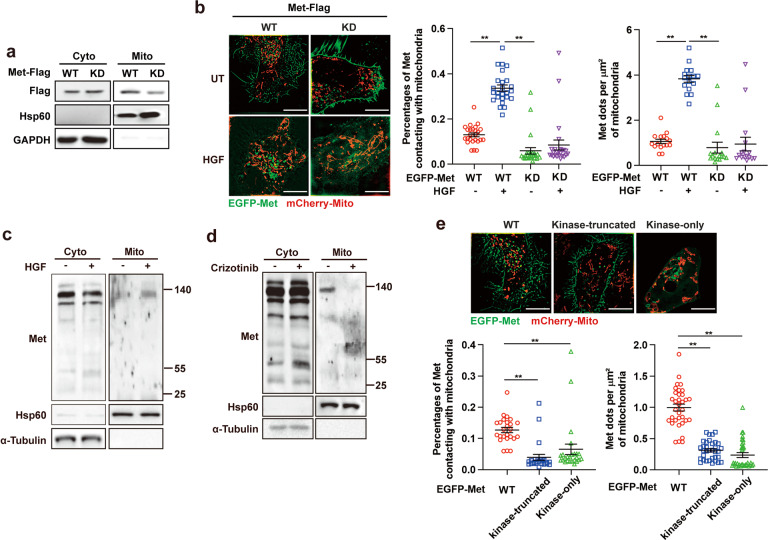
Kinase activity is required for mitochondrial trafficking of Met. **a** Immunoblot analysis of Flag-Met in mitochondrial and cytosolic fractions of *Met*^*-/*−^ Huh7 cells stably transfected with WT Flag-Met and KD Flag-Met. Mitochondria isolation markers, GAPDH (cytoplasmic) and Hsp60 (mitochondria) were used as controls. **b** *Met*^−*/*−^ HeLa cells stably expressing WT or KD EGFP–Met and mCherry–Mito were treated with HGF (100 ng/ml, 20 min) or not and then subjected to SIM live-cell imaging. Representative SIM images of mitochondrial localization of Met are shown. Scale bars, 10 µm. Quantification of percentages of Met contacting with mitochondria (*n* = 25 fields) and the number of Met dots docking at per µm^2^ of mitochondria (*n* = 16 fields). Error bars represent means ± SEM (**p < 0.01; Student’s *t* test). **c**, **d** Immunoblot analysis of intact and fragmented Flag-Met in mitochondrial and cytosolic fractions of Huh7 cells treated with HGF (100 ng/ml, 20 min) (**c**) or crizotinib (1 µM, 1 h) (**d**). Mitochondria isolation markers, α-Tubulin (cytoplasmic) and Hsp60 (mitochondria) were used as controls. **e** Representative SIM live-cell imaging of mitochondrial localization of Met in *Met*^*−/*−^ HeLa cells stably transfected with WT, kinase-truncated or kinase-only EGFP–Met. Scale bars, 10 µm. Quantification of percentages of Met contacting with mitochondria (n = 25 fields) and the number of Met dots docking at per µm^2^ of mitochondria (*n* = 34 fields). Error bars represent means ± SEM (**p < 0.01; Student’s *t* test)

We next investigated whether the kinase activity of Met was required for its mitochondrial localization. We re-expressed wild-type (WT) and kinase-dead (KD, K1110A)^39^ mutant Met in MET-deficient (*Met*^*−/−*^) Huh7 cells (generated using CRISPR/Cas9-mediated gene editing and previously characterized),^40^ and found that the expression levels of KD mutant Met were significantly lower than WT Met in the isolated mitochondrial fraction (Fig. 2a). Similar results were obtained under SIM in MET-deficient (*Met*^*−/−*^) HeLa cells re-expressed WT or KD Met tagged with an enhanced green fluorescent protein (EGFP–Met–WT or EGFP–Met–KD) and mitochondria-targeting mCherry fluorescent protein (mCherry–Mito). Approximately, 13% of EGFP–Met–WT was tethered with mitochondria, and HGF treatment markedly triggered Met mitochondrial localization (Fig. 2b), whereas a large percentage of EGFP–Met–KD was trapped in the cell membrane, with only approximately 7.3% of the entire fluorescence tethering with mitochondria, irrespective of HGF stimulation (Fig. 2b). Overall, these data confirm that Met kinase activity is required for its mitochondrial trafficking.

As Met protein was reported to be fragmented after HGF activation, we wondered whether intact or fragmented Met translocated to mitochondria. We observed that intact Met β chain (140 kDa) protein was expressed in the isolated mitochondrial fraction and the expression levels were significantly increased with HGF treatment (Fig. 2c) but decreased with crizotinib treatment (Fig. 2d), indicating that Met translocated into mitochondria in an intact form. In-depth analysis of Met fragments showed that the KT (1–1095 aa)^41^ type of Met was mainly maintained in the cell membrane, with a small percentage located in mitochondria, in agreement with the observations above (Fig. 2e). However, surprisingly, the kinase-only mutant Met (952–1048 aa),^39^ which lacked a transmembrane domain (856–952 aa),^42^ did not show mitochondrial localization and was diffusely distributed in the cytoplasm as well as vesicles (Fig. 2e), indicating that membrane carrying is a critical way for Met translocation.

### Met promotes mitochondrial fission relying on its activity

**Fig. 3:**
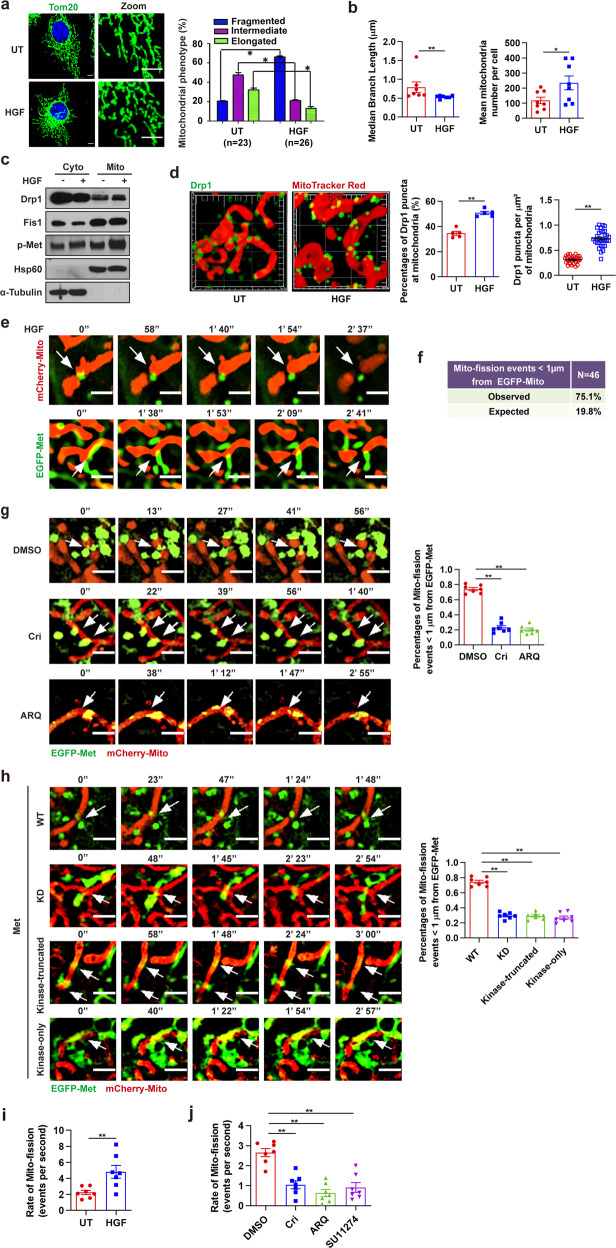
Mitochondrial fission is driven by Met kinase. **a** Huh7 cells were stimulated with HGF (100 ng/ml, 20 min) or not and then immunofluorescent stained for Met and Tom20. Percentages of cells with indicated mitochondrial morphologies were shown. Scale bars, 5 µm. Error bars represent means ± SEM (*n* represents the number of cells analyzed, *p < 0.05; Student’s *t* test). **b** Quantification of median branch length of individual mitochondria (*n* = 7 cells) and mean mitochondria number per cell (*n* = 8 cells) according to (**a**). Error bars represent means ± SEM (*p < 0.05, **p < 0.01; Student’s *t* test). **c** Immunoblot analysis of Drp1 and p-Met (Y1234-5) in mitochondrial and cytosolic fractions of Huh7 cells treated with HGF (100 ng/ml, 20 min). Mitochondria isolation markers, α-Tubulin (cytoplasmic) and Hsp60 (mitochondria) were used as controls. **d** Huh7 cells stimulated with HGF (100 ng/ml, 20 min) were immunofluorescent stained for Drp1 and mitochondria. Representative 3D SIM images of Drp1 assembly in mitochondria were shown. Images were captured in *Z*-stacks (frame, 10 µm × 10 µm; main calibration, 1 µm). Quantification of percentages of Drp1 puncta at mitochondria (*n* = 5 fields) and Drp1 puncta at per µm^2^ of mitochondria (*n* = 31 fields). Error bars represent means ± SEM (**p < 0.01; Student’s *t* test). **e** *Met*^*−/−*^ HeLa cells expressing WT EGFP-Met and mCherry–Mito were treated with HGF (100 ng/ml, 20 min). Representative time-lapse SIM live-cell imaging shows that Met contacts mitochondria at division sites before mitochondrial fission events happen (indicated with white arrows). Scale bars, 1 µm. Two fields were taken. **f** Quantification of percentages of mitochondrial division events that are marked by Met in HeLa cells transfected with WT EGFP-Met and mCherry–Mito (*n* = 46 events in 25 cells, *p* < 0.001, Fisher’s exact test). Quantified from SIM live-cell imaging described in (**e**). **g** Time-lapse SIM live-cell imaging shows Met contacts with mitochondria at the sites of mitochondrial division before fission (indicated with white arrows) in *Met*^*−/−*^ HeLa cells expressing WT EGFP-Met and mCherry–Mito with crizotinib (1 µM, 1 h) or ARQ-197 (5 µM, 1 h) treatments. Scale bars, 1 µm. Percentages of mitochondrial fission (Mito-fission) events marked by Met are quantified. Error bars represent means ± SEM (**p < 0.01; Student’s *t* test). Data are representative of seven independent experiments, 46 events per experiment. **h** Time-lapse N-SIM live-cell imaging of Met contacting mitochondria at the sites of mitochondrial division before fission (indicated with white arrows) in *Met*^*−/−*^ HeLa cells expressing WT, KD, kinase-truncated or kinase-only EGFP-Met and mCherry–Mito. Scale bars, 1 µm. Percentages of Mito-fission events marked by Met are quantified. Error bars represent means ± SEM (**p < 0.01; Student’s *t* test). Data are representative of seven independent experiments, 62 events per experiment. **i**, **j** Huh7 cells were treated with HGF (100 ng/ml, 20 min) (**i**), crizotinib (1 µM, 1 h), ARQ-197 (5 µM, 1 h) or SU11274 (1 µM, 1 h) (**j**), and then subjected to Live Cell Imaging. Mito-fission rates are quantified. Error bars represent means ± SEM (*n* = 7 cells, **p < 0.01; Student’s *t* test)

To determine the impact of Met kinase on mitochondrial function, we examined ATP production, calcium influx, and apoptosis in HCC cells treated with HGF. Little change was found in these aspects (Supplementary Fig. S1a–c). However, there were noticeable alterations in mitochondrial morphology: HGF triggered extensive mitochondrial fragmentation in HCC cells (Fig. 3a), resulting in decreased median branch length and increased numbers of individual mitochondria (Fig. 3b), without affecting mitochondrial activity or mitochondrial mass, as measured by tetramethylrhodamine ethyl ester and MitoTracker Green (Supplementary Fig. S1d), suggesting that Met might play an important role in the regulation of mitochondrial dynamics. Dynamin-like protein 1 (DnmL1; Drp1) is key mitochondrial fission executing protein that is recruited to form a ring at the mitochondrial outer membrane to contract the mitochondrial transverse diameter via ring constriction by GTPase activity.^43^ Dysregulation of Drp1 has been reported to contribute to tumor growth, metastasis, and chemoresistance.^29,35^ After HGF treatment, we assessed mitochondrial localization of Drp1 and found that HGF significantly stimulated Drp1 expression in isolated mitochondrial fractions (Fig. 3c). However, no significant differences were observed in either protein expression levels or mitochondrial localization of Mfn1, Mfn2, and OPA1, the mitochondrial fusion executing proteins (Supplementary Fig. S1e). We further determined the recruitment of Drp1 to mitochondria by anti-Drp1 antibody and Mito-Tracker Red using SIM-based immunofluorescent experiments and observed a significant increase in Drp1 puncta on mitochondria when the cells were treated with HGF (Fig. 3d). These data imply that Met may contribute to the mitochondrial fission program. To confirm the connection between Met and mitochondrial fission, we transfected *Met*^*−/−*^ HeLa cells with mitochondria-targeting mCherry fluorescent plasmid (mCherry-Mito) and WT, kinase dominated (KD), kinase-truncated (KT), or kinase-only Met tagged with EGFP. We subjected these cells to live SIM imaging to examine the actions of Met at mitochondria division sites. In WT cells treated with HGF, we noticed that mitochondrial fission sites were predominantly marked by Met protein before the event happened (white arrows) and the dynamic process of Met induced mitochondria division could be observed (Fig. 3e). As summarized, Met contacted mitochondria at 75.1% of the mitochondrial fission sites, which was significantly greater than that expected by random chance (19.8%; *p* < 0.001, Fisher’s exact test; Fig. 3f). When we treated the WT cells with crizotinib or ARQ-197, the mitochondrial fission sites marked by Met showed a substantial reduction, and the random contacting sites between Met and mitochondria did not undergo division (Fig. 3g). Moreover, KD Met, KT Met, or kinase-only Met mutants showed random contact with mitochondria, without promoting mitochondrial fission (Fig. 3h), suggesting that Met regulates mitochondrial fission through direct contact with mitochondria depending on its kinase activity. Finally, we used Live Cell Imaging to assess mitochondrial fission events. We observed that mitochondria underwent fission at an average rate of 2.67 events per second in Huh7 cells, and HGF stimulation elevated mitochondrial fission activities to an average rate of 4.33 events per second (Fig. 3i, Supplementary Fig. S1f and Supplementary Movies S2 and 3). Conversely, cells treated with crizotinib, ARQ-197, or SU11274 showed decay in mitochondrial fission activities (Fig. 3j and Supplementary Fig. S1g).

### Met interacts with mitochondrial fission protein Fis1 and triggers its tyrosine phosphorylation

**Fig. 4:**
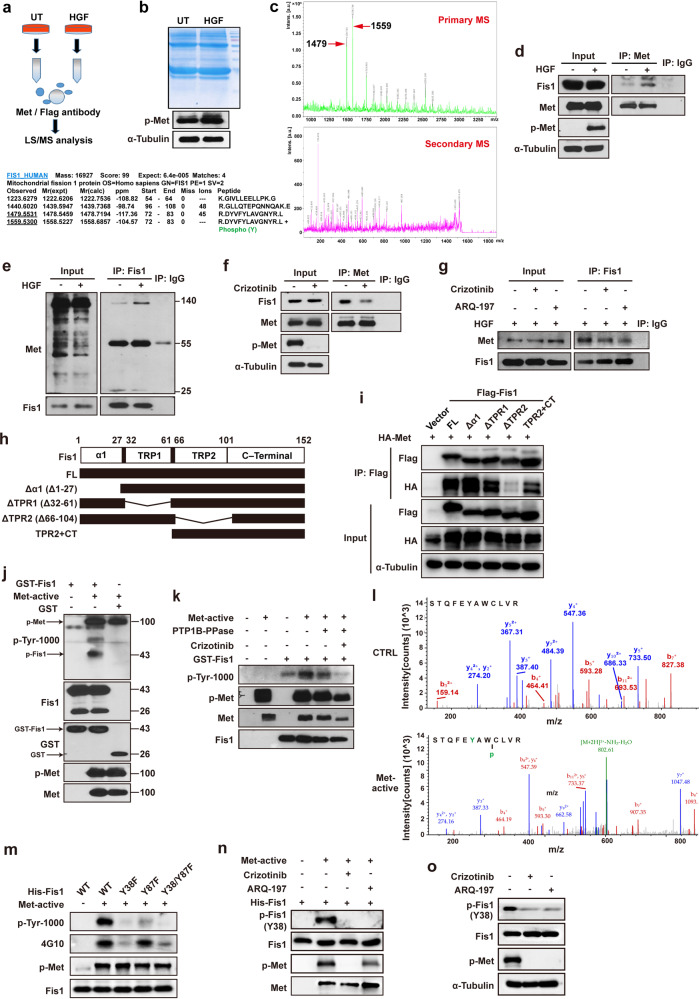
Met interacts with Fis1 and triggers its tyrosine phosphorylation. **a** The schematic diagram of proteomic analysis of Met co-binding proteins. **b** The comma blue stain of proteins co-IP with Met or normal IgG antibody. **c** Fis1 was detected in Met co-binding beads determined by MS analysis. **d**, **e** Huh7 cells were stimulated with HGF (100 ng/ml, 20 min) and then applied to IP assay with anti-Met antibody (**d**) or anti-Fis1 antibody (**e**). **f**, **g** Huh7 cells were treated with crizotinib (1 µM, 1 h) or ARQ-197 (5 µM, 1 h) and then applied to IP assay with anti-Met antibody (**f**) or anti-Fis1 antibody (**g**). **h** Schematic diagram of Fis1 truncations. The deleted regions are represented by lines. **i** HEK293T cells were transiently co-transfected with the indicated plasmids for 48 h. Cells were lysed and immunoprecipitated with anti-Flag antibody. Co-immunoprecipitated HA-tagged Met was detected by immunoblotting. **j** Purified GST–Fis1 fusion protein was incubated with recombinant activated Met kinase for 30 min in the kinase buffer with ATP, and then subjected to WB. **k** Purified GST–Fis1 fusion protein was incubated with activated Met kinase in the presence of crizotinib or protein–tyrosine phosphatase (PTP1B) for 1 h, and the phosphorylation levels of Fis1 were detected with WB. **l** Purified His–Fis1 fusion protein was incubated with activated Met for 30 min, and then subjected to LC–MS analysis to detect phosphorylated tyrosine sites. **m** Purified Fis1 (WT), Fis1 (Y38F), Fis1 (Y87F), and Fis1 (Y38/87F) fusion proteins were incubated with activated Met for 30 min. Two kinds of anti-phosphorylation antibodies (p-Tyr-1000 and p-Tyr 4G10) detecting total phosphotyrosine levels were used in WB. **n** His–Fis1 fusion protein was incubated with Met kinase for 30 min in the presence of crizotinib (1 µM, 1 h) or ARQ-197 (5 µM, 1 h) or not, and the phosphorylation levels of Fis1 were determined with specific Fis1 pY38 antibody. **o** Huh7 cells were treated with crizotinib (1 µM) or ARQ-197 (5 µM) for 1 h, and the expression levels of p-Fis1 (Y38) was determined with specific Fis1 pY38 antibody

To investigate the mechanism underlying Met-mediated mitochondrial fission, we transiently expressed Flag-tagged Met in HEK293T cells, pulled Met protein down with anti-Flag beads, and then detected the global protein interactome of Met through liquid chromatography–mass spectrometry (LC–MS). We identified mitochondrial fission protein (Fis1) interacting directly with Met (Fig. 4a–c). Met interaction with Fis1 was confirmed based on protein–protein interactome (PPI) by LC–MS (Supplementary Fig. S2a, b). The interaction between Met and Fis1 was further verified through co-immunoprecipitation (co-IP) experiments. It was found that Fis1 bound to the full-length β subunit of Met kinase (140 kDa) at endogenous levels, and the binding increased when stimulated with HGF (Fig. 4d, e) but attenuated when treated with Met kinase inhibitors (Fig. 4f, g). Met interaction with Fis1 could also occur in other types of human cancer cells like HCC1806 breast cancer cells (Supplementary Fig. S2c) and HT29 colon cancer cells (Supplementary Fig. S2d), which was analogously enhanced by HGF but diminished by crizotinib. However, in LO2 normal liver cells, the interaction between Met and Fis1 was weak and could not be regulated by Met kinase, indicative of the unique role of Met interaction with Fis1 in cancer (Supplementary Fig. S2e). We also examined the interaction of Met with other proteins involved in mitochondrial dynamics. Weak interactions between exogenous Met and Drp1 or Mfn2 were observed in Huh7 cells (Supplementary Fig. S2f). No interactions were detected between exogenous Met and Mfn1, OPA1, Mff, Mid49, or Mid51 (Supplementary Fig. S2f). In contrast, Met robustly interacted with Fis1 at exogenous levels and the interaction was enhanced with HGF treatment (Supplementary Fig. S2f). To further delineate the domain of Fis1 responsible for the interaction between Met and Fis1, we assembled deletion constructs of Fis1 (Fig. 4h). We transiently expressed HA-tagged Met and Flag-tagged Fis1 deletion constructs in 293 T cells and assessed the association of Fis1 truncations with Met by Co-IP. Deletion of the TPR2 domain significantly decreased Met binding, whereas the constructs containing TPR2 were able to bind Met, indicating that the TPR2 domain of Fis1 was necessary for the interaction between Fis1 and Met (Fig. 4i).

We then tested whether Met kinase would catalyze the phosphorylation of Fis1 tyrosine residue with an in vitro kinase assay. We verified that the total phosphorylation of Fis1 was promoted by Met kinase but attenuated with Met kinase inhibition or phosphatase addition (Fig. 4j, k and Supplementary Fig. S2g), suggesting that Met kinase triggered tyrosine phosphorylation of Fis1 directly. To identify Tyr phosphorylation site(s) of Fis1, we used mass spectrometry to analyze purified His–Fis1 fusion protein which had been stimulated with activated Met in vitro. We identified two peptides phosphorylated at Y38 and Y87 of Fis1 (Fig. 4l). For further verification, we generated Fis1 Y38F or Y87F mutants (tyrosine residues mutated to nonphosphorylated phenylalanine) and test their effects on the phosphorylation of Fis1 in an in vitro kinase assay. As shown, phosphorylation was substantially decreased in Fis1 Y38F mutant but not in the Y87F mutant, compared to the WT form, indicating that Y38 was the major Met phosphorylation site (Fig. 4m and Supplementary Fig. S2h). To reconfirm this result, we generated an antibody to specifically detect pY38. Either in an in vitro kinase assay or in an endogenous experiment using Huh7 cell line, treatment with Met inhibitors largely diminished phosphorylation of Fis1 at Y38 (Fig. 4n, o), confirming that Met phosphorylated Fis1 at Y38 directly.

### Phosphorylation of Fis1 at Y38 triggers Drp1 assembly at mitochondria and promotes mitochondrial fission

Fis1 was reported to recruit Drp1 to mitochondria and facilitate mitochondrial fission. Structurally, two regions of Fis1 have been previously implicated in Drp1 recruitment: an autoinhibitory N-terminal “arm”(NTE) and a concave surface formed by Fig evolutionarily conserved residues in the tetratricopeptide repeat (TPR) domain. Whereas NTE and TPR domains are insufficient to regulate Drp1 binding to Fis1, and there may be the mitochondrial outer membrane or a covalent modification, such as phosphorylation.^44^ Therefore, we attempted to determine that whether Met-mediated phosphorylation of Y38 of Fis1 would affect Drp1 mitochondrial assembly and mediate mitochondrial fission.

**Fig. 5:**
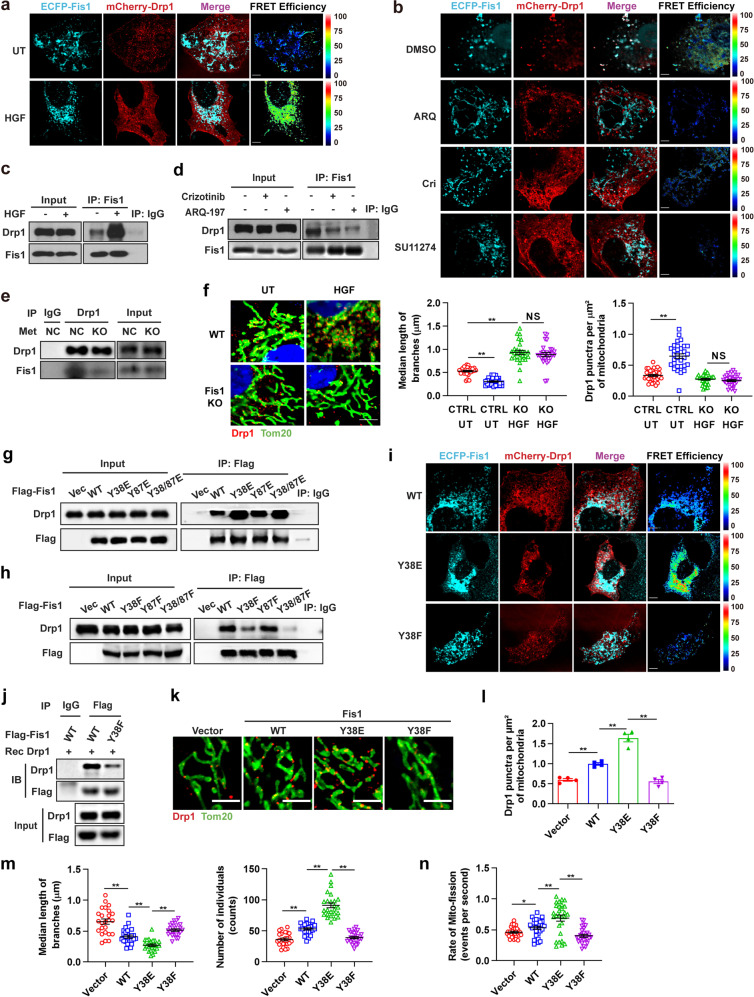
Y38 Phosphorylation of Fis1 facilitates Drp1 assembly to mitochondria and promotes mitochondrial fission. **a** Representative confocal images of HeLa cells expressing FRET pairs (ECFP–Fis1 and mCherry–Drp1) demonstrating preferentially increased SE-FRET signals with the treatment of HGF (100 ng/ml, 20 min). Scale bars, 5 µm. **b** Representative confocal images of HeLa cells expressing FRET pairs (ECFP–Fis1 and mCherry–Drp1) demonstrating decreased SE-FRET signals with the treatment of crizotinib (1 µM, 1 h), ARQ-197 (5 µM, 1 h) or SU11274 (1 µM, 1 h). Scale bars, 5 µm. **c**, **d** Huh7 cells were stimulated with HGF (100 ng/ml, 20 min) (**c**) or treated with crizotinib (1 µM, 1 h) or ARQ-197 (5 µM, 1 h) (**d**) and then applied to IP assay with anti-Fis1 antibody. **e** Met knockout Huh7 cells and their control were applied to IP assay with anti-Drp1 antibody. **f** Representative confocal images of mitochondrial morphology in WT or *Fis1*^*−/−*^ Huh7 cells treated with HGF (100 ng/ml, 20 min) or not. Cells were stained for Tom20 and Drp1. Scale bars, 5 µm. Quantification of branch length of mitochondria (*n* = 27 cells) and Drp1 puncta docking at mitochondria (n = 31 cells). Error bars represent means ± SEM (*p < 0.05, NS denotes no statistical significance; Student’s *t* test). **g**, **h** *Fis1*^*−/−*^ Huh7 cells were transiently transfected with empty vector, WT Fis1-Flag, and indicated Fis1 phosphomimetic mutants (**g**) or nonphosphorylatable mutants (**h**) tagged with Flag, and then applied to IP assay with anti-Flag antibody. **i** Representative confocal images of HeLa cells expressing FRET pairs (mCherry–Drp1 and ECFP–Fis1 WT, ECFP–Fis1 Y38E, or ECFP–Fis1 Y38F) demonstrating corresponding SE-FRET signal. Scale bars, 5 µm. **j** Western blot detection of the binding between recombinant Drp1 and Fis1 protein in an in vitro pull-down assay. WT or Y38F Fis1 was immunoprecipitated with Flag beads from Huh7 cells. **k** Representative confocal images of mitochondrial morphology in *Fis1*^*−/−*^ Huh7 cells transfected with empty vector, Fis1 WT, Fis1 Y38E mutant, or Fis1 Y38F mutant. Cells were stained for Tom20 and Drp1. Scale bars, 2 µm. **l** Quantification of Drp1 puncta locating at per µm^2^ of mitochondria according to (**k**). Error bars represent means ± SEM (*n* = 4 cells, **p < 0.01; Student’s *t* test). **m** Quantification of branch length of individual mitochondria and mitochondria number per cell according to (**k**). Error bars represent means ± SEM (*n* = 25 cells, **p < 0.01; Student’s *t* test). **n** Quantification of mito-fission rates of *Fis1*^*−/−*^ Huh7 cells expressing empty vector, Fis1 WT, Fis1 Y38E mutant, or Fis1 Y38F mutant through Live Cell Imaging. Error bars represent means ± SEM (*n* = 28 cells, *p < 0.05, **p < 0.01; Student’s *t* test)

We first evaluated the effect of Met kinase on the interaction between Fis1 and Drp1. An association between ECFP-Fis1 and mCherry-Drp1 in HeLa cells could be observed through a sensitized emission fluorescence resonance energy transfer (SE-FRET) assay and the association was significantly enhanced with HGF stimulation (Fig. 5a) but attenuated by Met kinase inhibitors (Fig. 5b). A similar interaction pattern at endogenous levels was obtained in Huh7 cells through co-IP experiments (Fig. 5c, d). Furthermore, ablation of Met extensively attenuated the interaction between Fis1 and Drp1 (Fig. 5e), revealing that Fis1 interacted with Drp1 relying on Met kinase. We next examined the effect of Met on Drp1 mitochondrial recruitment in Huh7 cells. Using confocal microscopy, we found that HGF stimulation led to mitochondrial fragmentation, consistent with previous observations, and Fis1 knockout diminished HGF-induced mitochondrial fission (Fig. 5f). Moreover, we found that HGF significantly stimulated mitochondrial assembly of Drp1 and the effect was thoroughly blocked by Fis1 knockout (Fig. 5f). Consistently, in a subcellular fractionation assay, we found that *Met*^*−/−*^ Huh7 cells re-expressed with KD-Met exhibited reduced mitochondrial expression of Drp1 compared to cells re-expressed with WT-Met (Supplementary Fig. S3a). In conclusion, these data establish that Met kinase promotes Drp1 mitochondrial assembly and facilitates mitochondrial fission through Fis1 protein.

To explore whether Met-induced phosphorylation of Fis1 at Y38 or Y87 affected its ability to recruit Drp1, either or both of the Y38 and Y87 tyrosine residues of Fis1 were mutated to phosphomimetic glutamate (Glu, E) or nonphosphorylatable phenylalanine (Phe, F). When re-expressed in *Fis1*^*−/−*^ cells, Y38E mutant Fis1 showed significantly enhanced association with Drp1 compared to WT Fis1, whereas the Y38F mutant exhibited only a weak interaction, as showed by SE-FRET assay in HeLa cells and co-IP experiments in Huh7 cells (Fig. 5g–i). Meanwhile, Y87E mutant, Y87F mutant and WT Fis1 exhibited comparable ability to associate with Drp1 (Fig. 5g, h), supporting the notion that Met phosphorylated Fis1 at Y38 but not Y87. To further illustrate the association between phosphorylated Fis1 and Drp1, we used an in vitro pull-down assay to test the binding of Drp1 recombinant protein and WT or nonphosphorylatable mutant (Y38F) Fis1, which were immunoprecipitated with Flag beads from Huh7 cell lysates (Fig. 5j). It was found that WT Fis1 bound to Drp1 recombinant protein intensively, whereas the binding was markedly repressed when Fis1 was nonphosphorylatablely mutant, indicating that Fis1 interacted directly with Drp1 relying on the phosphorylation of Y38. These data suggest that phosphorylation of Y38 of Fis1 is critical for its ability to recruit Drp1.

We next set out to investigate whether Fis1 pY38 mediated Drp1 recruitment triggered Drp1 assembly at mitochondria and promoted mitochondrial fission. Cells expressing Y38E mutant Fis1 exhibited augmented Drp1 assembly at mitochondrial fission sites and more fragmented mitochondrial morphology compared to cells expressing WT Fis1, while the Y38F mutant exhibited the weakest effect (Fig. 5k-m). Mff and MIEF1/2 (MiD51/49) are another set of mitochondrial receptors that serve to mediate the recruitment of Drp1 to mitochondria.^45,46^ In contrast to the dynamic binding between phosphorylated Fis1 and Drp1 relying on Met kinase, interactions between Drp1 and Mff or MiD51/49 stayed consistent irrespective of the phosphorylation state of Fis1, indicating that Fis1 pY38 promoted Drp1 mitochondrial recruitment independent of other receptors (Supplementary Fig. S3b). Fis1 has been reported to drive fragmentation of the mitochondrial network by binding to Mfn1, Mfn2, and OPA1 and thus blocking the fusion machinery.^47^ In our study, in line with the previous report, WT Fis1 is associated with Mfn1, Mfn2, and OPA1. However, when we mutated Y38 of Fis1 to a nonphosphorylatable form, no decrease was seen in the interactions. By contrast, the interaction between Y38F Fis1 and Drp1 showed a noticeable decline compared to WT Fis1, demonstrating that Fis1 pY38 promoted mitochondrial fragmentation specifically through Drp1 (Supplementary Fig. S3c). To further corroborate Fis1 pY38 mediated mitochondrial fission, we used Live Cell Imaging to assess mitochondrial fission incidence of Huh7 cells. It was showed that re-expression of both WT Fis1 and the Y38E mutant but not the Y38F mutant rescued mitochondrial fission incidence in *Fis1*^*−/−*^ Huh7 cells, and the Y38E mutant led to even higher mitochondrial fission incidence than the WT (Fig. 5n, Supplementary Fig. S3d and Supplementary Movie S4–7).

### Met mediated Fis1 Y38 phosphorylation facilitates cell metastasis in vitro and in vivo

**Fig. 6:**
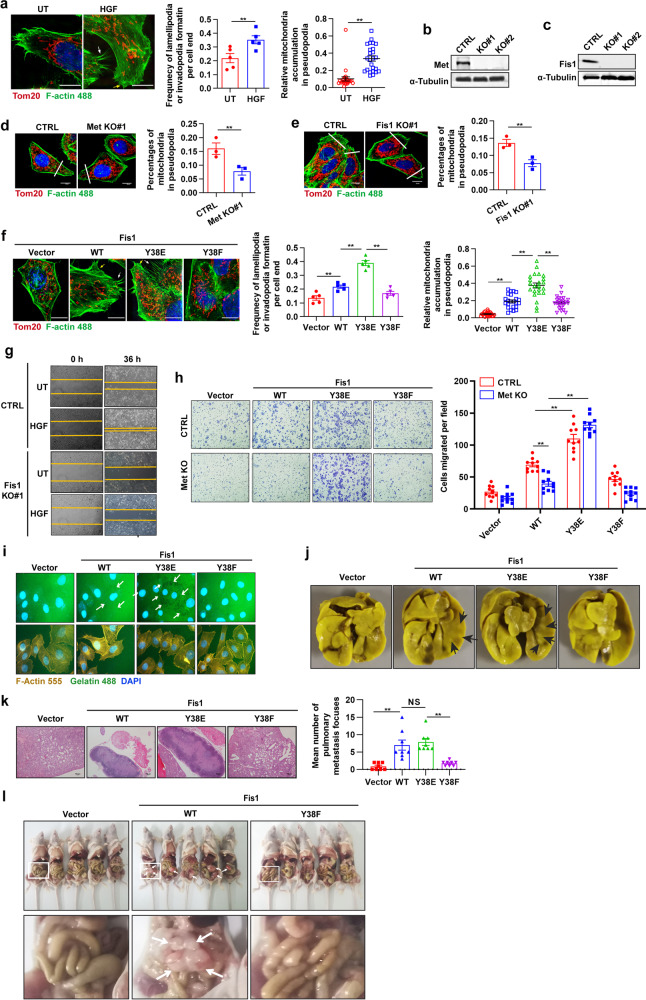
Met-mediated Fis1 Y38 phosphorylation facilitates cell metastasis in vitro and in vivo. **a** Representative SIM images of lamellipodia (yellow arrows) and invadopodia (white arrows) forming at the leading edge of Huh7 cells treated with HGF (100 ng/ml, 20 min). Mitochondria were stained with anti-Tom20 antibody and F-actin was stained with phalloidine. Scale bars, 10 µm. Quantification of the frequency of lamellipodia and (or) invadopodia formation of each cell end (*n* = 5 cells) and relative abundance of mitochondria in lamellipodia or invadopodia region (*n* = 25 cells). Error bars represent means ± SEM (**p < 0.01; Student’s *t* test). **b**, **c** Immunoblot analysis of Met knockout efficacy (**b**) or Fis1 knockout efficacy (**c**) in Huh7 cells using CRISPR-Cas9 technology. **d**, **e** Representative images of lamellipodia and (or) invadopodia at the leading edge of *Met*^*−/−*^ (**d**) or *Fis1*^*−/−*^ (**e**) Huh7 cells. Quantification of relative abundance of mitochondria in the lamellipodia or invadopodia region. Scale bars, 10 µm. Error bars represent means ± SEM (*n* = 3 cells, **p < 0.01; Student’s *t* test). **f** Representative SIM images of lamellipodia (yellow arrows) and invadopodia (white arrows) forming at the leading edge of *Fis1*^*−/−*^ Huh7 cells re-expressed with indicated plasmids. Mitochondria were stained with anti-Tom20 antibody and F-actin was stained with phalloidine. Scale bars, 10 µm. Quantification of the frequency of lamellipodia and (or) invadopodia formation of each cell end (*n* = 5 cells) and relative abundance of mitochondria in lamellipodia or invadopodia region (*n* = 23 cells). Error bars represent means ± SEM (**p < 0.01; Student’s *t* test). **g** Representative images of wound healing assay in WT or *Fis1*^*−/−*^ Huh7 cells stimulated with HGF (100 ng/ml) for indicated time or not, implying the migration ability. **h** *Fis1*^*−/−*^ Huh7 cells with Met knocked out or not were transfected with indicated plasmids. Transwell assay was applied to examine the migration ability of these cells. Quantification of cells migrated per field. Error bars represent means ± SEM (*n* = 10 fields, **p < 0.01; Student’s *t* test). **i** Representative images of cytoskeleton morphology in *Fis1*^*−/−*^ huh7 cells transfected with indicated plasmids. Cells were stained with F-actin 555 and incubated in an extracellular matrix substituted with F-488 conjugated gelatin. The white arrows indicate the gelatin degraded by cancer cells (dark spots). **j**, **k** *Fis1*^*−/−*^ Huh7 cells re-expressed with indicated plasmids were injected to nude mice for 1 × 10^6^/mouse through tail vein, pulmonary metastasis was tested after 28 days. Representative nodules on the pulmonary surface after picric acid staining for 6 h (**j**). Representative images of lung histological sections with HE staining (**k**, left). Scale bars, 100 µm. Quantification of the mean number of lung metastases per mice (**k**, right). Error bars represent means ± SEM (*n* = 8 mice, **p < 0.01, NS denotes no statistical significance; Student’s *t* test). **l** Nude mice were intraperitoneally inoculated with *Fis1*^*−/−*^ Huh7 cells re-expressed with indicated plasmids (n = 5 mice) and tumor nodes in the abdominal cavity were observed after 21 days. The white arrows indicate the tumor nodes on the peritoneum. The zoomed images are shown below

We next investigated that whether Fis1 pY38 promoted cellular lamellipodia or invadopodia formation, which was resulted from mitochondrial fission-based mitochondria redistribution and necessary for cell migration. Based on SIM, we found that HGF stimulation promoted lamellipodia (Fig. 6a, yellow arrow) or invadopodia (Fig. 6a, white arrow) formation at the leading edge of cells as well as mitochondrial accumulation in the pseudopodia area. Knocking out either Met or Fis1 resulted in diminished pseudopodia formation and mitochondrial accumulation in the pseudopodia area (Fig. 6b–e). Re-expression of Y38E-mutant Fis1 led to the higher frequency of lamellipodia or invadopodia formation as well as higher percentages of mitochondrial accumulation in the pseudopodia area compared to WT, while the Y38F mutant exhibited the least (Fig. 6f). These data suggest that Met-mediated Y38 phosphorylation of Fis1 facilitates lamellipodia or invadopodia formation through mitochondrial redistribution.

By the use of wound healing assay (Supplementary Fig. S4a), transwell assay (Supplementary Fig. S4b), and extracellular matrix (ECM) degradation assay (Supplementary Fig. S4c, d), we confirmed that Met kinase facilitated cell migration and invasion, consistent with previous reports.^48^ Innovatively, we found that Fis1 knockout led to a blockade of cell migration and invasion, which were supposed to be promoted by HGF (Fig. 6g and Supplementary Fig. S4e, f), while knocking down another pro-fission protein MFF by siRNA did not exhibit similar effect (Supplementary Fig. S4g), suggesting a pivotal role of Fis1 in Met induced cell metastasis. We also evaluated the effect of Fis1 on cell metastasis in a pulmonary metastasis model, which was established by injecting Huh7 cells via the tail vein, and found that compared to the WT cells, Fis1^*−/−*^ Huh7 cells could hardly form pulmonary metastasis (Supplementary Fig. S4h, i).

We then asked whether Met-mediated Fis1 pY38 promoted cell migration and invasion. To test this, we re-expressed WT, Y38E-mutant or Y38F-mutant Fis1 in *Fis1*^*−/−*^ Huh7 cells with Met knock out or not. As expected, re-expression of the Y38E mutant enhanced the migration ability compared to WT Fis1, whereas the Y38F mutant showed no difference (Fig. 6h). Knocking out Met diminished the migration of cells expressing WT Fis1, but it did not affect cells expressing Y38F-mutant or Y38E-mutant Fis1 (Fig. 6h). We observed similar results in an ECM degradation assay (Fig. 6i). In addition, we examined the effect of Fis1 pY38 on cell growth, cell cycle, apoptosis, and drug resistance to olaparib or sorafenib in HCC cells, and found no significant differences in these aspects (Supplementary Fig. S4j–m). In line with the results in vitro, in a nude mice pulmonary metastasis model, cells expressing WT Fis1 had restored metastatic foci forming ability while cells expressing the Y38F mutant displayed hampered metastatic foci formation (Fig. 6j, k). Met kinase of WT cells was forcefully activated in vivo, which was perhaps due to the HGF ligands secreted from stromal cells,^49^ thus as showed, WT cells and Y38E cells displayed no differences in pulmonary metastatic ability (Fig. 6j, k). We further established a mouse model mimicking peritoneal metastasis by intraperitoneally injecting Huh7 cells. We revealed that cells expressed with WT Fis1 quickly migrated to the mesenterium or the omentum majus, while no peritoneum metastasis was seen in Y38F cells (Fig. 6l). These data illustrate that Met-mediated Fis1 pY38 is specifically essential for HCC cell metastasis. In view of the crucial role of the Met–Fis1–Drp1 axis in mitochondrial fission and cell metastasis, we tried to examine the effect of combining mitochondrial fission inhibitor (Mdivi-1) and Met inhibitor (Crizotinib). Both mdivi-1 and crizotinib efficiently inhibited migration and invasion of Huh7 cells, and the combination exhibited the strongest effect, suggesting a promising therapy for HCC metastasis (Supplementary Fig. S4n, l).

### Expression levels of p-Fis1 and HGF are positively correlated in HCC patients and both indicate a dismal prognosis

**Fig. 7:**
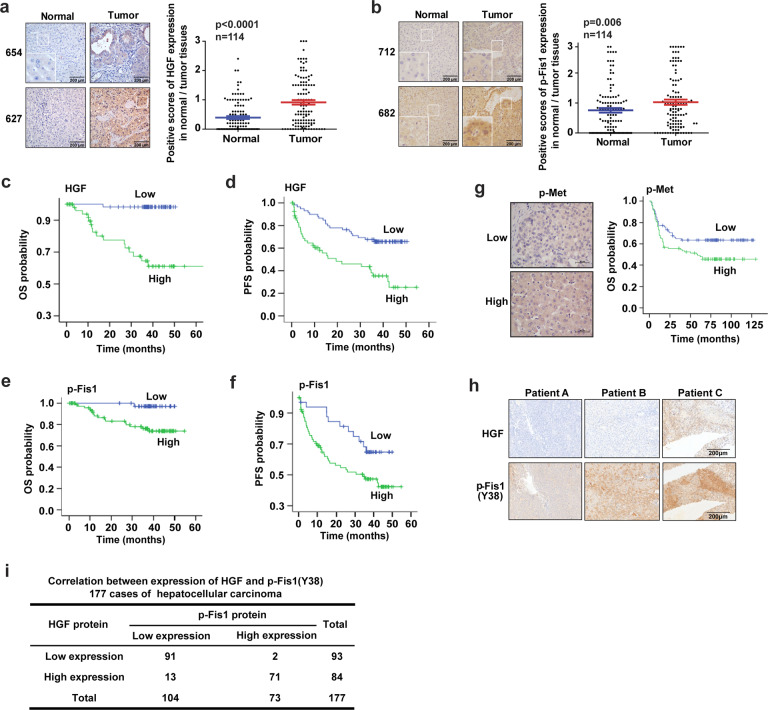
Clinical relevance of p-Fis1 and HGF in HCC. **a**, **b** Representative images of HGF (**a**) and p-Fis1 (Y38) (**b**) staining in HCC patient specimens. Scale bars, 200 µm. The HGF or p-Fis1 (Y38) scores are shown. The student’s *t* test was used for statistical analysis. **c**, **d** Kaplan–Meier overall survival (OS) (**c**) and progression-free survival (PFS) (**d**) curves for HCC patients according to HGF expression in tumor tissues, the log-rank test was used to determine significance. **e**, **f** Kaplan–Meier overall survival (OS) (**e**) and progression-free survival (PFS) (**f**) curves for HCC patients according to p-Fis1 (Y38) expression in tumor tissues, the log-rank test was used to determine significance. **g** Representative images of p-Met staining in HCC patient specimens (upper). Scale bars, 50 µm. Kaplan–Meier overall survival (OS) for HCC patients according to p-Met expression in tumor tissues, the log-rank test was used to determine significance (lower). **h**, **i** Representative images of HGF and p-Fis1 (Y38) staining in HCC patient specimens (**h**). The Spearman correlation test or Cox regression analysis was used to validating the correlation between HGF and p-Fis1 (Y38), *p* value was caculated (**i**)

To evaluate the clinical relevance of our findings, 115 pairs of HCC tumorous and corresponding adjacent non-tumorous tissue samples were obtained from patients who underwent curative resection. Immunohistochemistry analysis showed that p-Fis1 and HGF were expressed at higher levels in tumor tissues compared to adjacent non-tumorous tissue (Fig. 7a, b). Survival analysis of the 115 HCC cases indicated that patients with high p-Fis1 and HGF protein levels had much more negative prognoses (recurrence, metastasis, or death) (Logrank *p* < 0.01; Fig. 7c–f). High expression of HGF or p-Fis1 (Y38) was correlated with shorter tumor-free interval; the median tumor-free interval in patients with low HGF expression was 59.17 months, while in patients with high HGF expression, it was only 49.19 months (Fig. 7d); the median tumor-free interval in patients with low p-Fis1 (Y38) expression was 48.29 months, while in patients with high p-Fis1 (Y38) expression, it was only 28.58 months (Fig. 7f). Similarly, in an HCC tissue microarray containing 177 cases, immunohistochemical (IHC) staining and Kaplan–Meier survival analysis of p-Met revealed that the HCC patients with a high expression of p-Met had a shortened OS compared to that with low expression (Logrank *p* = 0.034; Fig. 7g). Next, we analyzed the correlation between p-Fis1 (Y38) and HGF in the HCC tissue microarray. We found that 93 cases (52.5%) showed low expression of HGF, and 104 cases (58.8%) showed low expression of p-Fis1. Ninety-one cases (51.4%) showed negative expression of p-Fis1 and HGF meanwhile; 71 cases (40.1%) showed positive expression of p-Fis1 and HGF meanwhile. Spearman correlation analysis revealed that the expression of p-Fis1 and HGF were positively correlated (*r* = 0.836, *p* < 0.001) (Fig. 7h, i). Together, our findings defined a central role of Met in activating Fis1 by tyrosine phosphorylation and promoting mitochondrial fission to facilitate HCC metastasis, suggesting its use in novel strategies to inhibit tumor recurrence and metastasis.

## Discussion

Reorganization of the cytoskeleton is crucial for the motility of tumor cells and requires the consumption of a large amount of ATP. In terms of energy metabolism, tumor cells are abnormal in their abundance of biosynthesis during proliferation, which requires a high level of aerobic glycolysis.^34^ However, in cells undergoing migration or invasion, extra ATP synthesized mainly in mitochondria is required for cytoskeletal rearrangement. Zhao et al. reported that in invasive breast cancer cells, mitochondrial fission regulates the invasion and migration of breast cancer cells by redistribution of mitochondria in the leading edge of cells to fuel lamellipodial formation.^35^ However, the mechanism under which mitochondrial fission is initiated and monitored under the condition of cell metastasis is not clearly understood. Our study elucidated a new mechanism by which Met, a tyrosine kinase receptor, directly regulates fission and promotes liver cancer metastasis.

Met shares many structural and functional similarities with EGFR. Both are protein tyrosine kinase receptors and can promote cell proliferation and tissue renewal under physiological conditions. Overexpression and activation of Met have been reported frequently in advanced non-small cell lung cancers with resistance to EGFR inhibitors or antibodies.^50^ Therefore, Met may play an important role in drug resistance and metastasis rather than EGFR. We found that Met could be directly located in mitochondria and regulate mitochondrial fission through kinase activity. HGF stimulation enhanced mitochondrial fission, while Met inhibitor treatment significantly suppressed mitochondrial fission. The invasion and migration ability is in line with Met kinase-regulated mitochondrial fission, as knocking out Fis1 resulted in hampered mitochondrial fission and cell invasion and migration, which would be prompted by HGF stimulation. Therefore, through the Met tyrosine kinase receptor, the signal carried by extracellular HGF is thus transmitted to mitochondria and promotes the metastasis of tumors by activating mitochondrial fission. Our work provides a novel perspective to better understand the tyrosine kinase receptor Met and may be helpful for targeting therapeutics.

Fis1 is an important recruiting protein on the outer membrane of mitochondria. In prokaryotic cells, Fis1 assembles with Drp1 on the outer mitochondrial membrane to form a Drp1 ring and initiates mitochondrial fission by ring contraction and scission. In eukaryotic cells, the component of the mitochondrial fission complex is much more intricate and involves more members and regulatory mechanisms.^51^ It is generally accepted that Fis1 participates in the recruitment of Drp1 to mitochondria either directly or through the regulation of other structural proteins, such as MFF and Mid49/51.^52^ It has been reported that overexpression of Fis1 promotes the occurrence of mitochondrial fission in either normal or cancer cells.^30^ Interestingly, in some cases, Fis1 is not necessary for mitochondrial fission in eukaryotic cells, and the cells with Fis1 knockout can replicate and proliferate without obvious changes in mitochondrial morphology.^53^ According to these findings, we infer that under static conditions, the role of Fis1 in mediating mitochondrial fission may be substituted by other components; however, under certain circumstances, such as tumor cells undergoing migration or invasion with high energy requirements, Fis1 can be activated and strongly promotes mitochondrial fission for special energy demands. Our studies demonstrate that Met phosphorylates Y38 of Fis1 in vitro and in vivo, and the phosphorylation of Fis1 activates its ability to recruit Drp1 and facilitate mitochondrial fission. HCC cells with Y38E (phosphomimetic) mutant Fis1 exhibited enhanced migration and invasion in vitro and in vivo. It has been reported that the Fis1 N-terminal region (1–20 amino acids) can form an autoinhibitory structure, which blocks Drp1 recruitment. Y38 phosphorylation could abolish Fis1 N-terminal negative structure so that Drp1 binding increased with Fis1.^44^ By IHC staining of liver cancer tissues, we also confirmed that p-Fis1 was a good predictor of recurrence and overall survival of patients with HCC, and high expression of p-Fis1, as well as HGF, was positively associated with poor prognosis. Therefore, our work provides closer insight into the indeterminate role of Fis1 protein, illuminating that Fis1 is particularly activated in tumor cells that are migrating and invading with large energy demands.

HGF/c-Met signaling has been reported to be involved in tumor metastasis by activating its downstream effector components. Signaling by the RAS/MAPK and PI3K/AKT pathways reaches the nucleus to affect gene expression related to angiogenesis, invasion, and metastasis.^54,55^ Cytoplasmic signaling cascades mediated by RAC1/CDC42 and PAK elicit cytoskeletal changes for cell motility.^56,57^ Signals through the RAP1 and RAC1/CDC42 pathways reach the plasma membrane and control cadherin and integrin adhesion molecules and thereby affect cell migration.^57,58^ Our study further improved the downstream regulation network of Met in tumor metastasis. As wound healing assay showed, HGF induced enhanced cell migration was almost blocked by Fis1 knockout (Fig. 6g), and in a nude mice pulmonary metastasis model, Met mediated Fis1 pY38 was necessary for metastatic foci forming (Fig. 6j, k), uncovering an irreplaceable role of Fis1 pathway in Met-mediated cell metastasis.

In our study, we found that mdivi-1, a mitochondrial fission inhibitor, which acted on both Drp1 and Dynamin I, synergized with Met to constrain cell migration and invasion. P110 has been reported as a Drp1-specific inhibitor to impede mitochondrial fragmentation by inhibiting Drp1 enzyme activity as well as blocking Drp1/Fis1 interaction.^59^ Accordingly, based on our finding, compared to mdivi-1, P110 combination with Met inhibitor may be a more individualized and potential therapy to prevent metastasis in HCC patients.

Using mass spectrometry analysis, we found that, in fact, Fis1 as a binding protein, in addition to Drp1, also interacts with a large number of proteins involved in the regulation of mitochondrial movement, mitochondria and endoplasmic reticulum contacts, the association of mitochondria and microfilaments, as well as a large fraction of proteins involved in membrane remodeling and vesicle secretion. Hence, apart from mitochondrial motility, HGF-mediated phosphorylation and activation of Fis1 may play a broader role in promoting HCC metastasis, and this needs to be confirmed and further explored.

In this study, we verified that mitochondrial fission is indispensable for HCC metastasis. In the microenvironment of HCC, HGF plays a pivotal role in the migration and invasion of cancer cells through activation of Met kinase acting on mitochondrial fission directly. This study provides a theoretical basis for the application of Met-targeted inhibitors in clinical trials of HCC. We found that Fis1 is a new important downstream target regulated by Met kinase. We elucidated for the first time the biological significance of Met in regulating the phosphorylation of Y38 of Fis1 in metastatic liver cancer cells, providing a new predictor for the relapse and prognosis of patients with liver cancer. In addition, we provided a new therapeutic strategy to prevent metastasis of liver cancer, either by blocking mitochondrial fission alone or combined with Met kinase inhibitors.

## Materials and methods

### Plasmids and compounds

To construct pLYS1-mCherry-Mito, mCherry cDNA was subcloned from pmCherry-C1 Vector (Clontech Laboratories, #632524) and inserted into pLYS1-FLAG-MitoGFP-HA (Addgene, #50057)^60^ with EGFP encoding sequence removed. PCDH-puro-Met-Flag, PCDH-puro-Met-KD-Flag were gifts from Mien-Chie Hung’s Lab. Human full-length Met cDNA was subcloned into pmCherry-C1 Vector and pEGFP-C1 Vector (Clontech Laboratories, #6084-1) to construct mCherry-Met and EGFP-Met. Met-KD cDNA was subcloned into pEGFP-C1 Vector to construct EGFP-Met-KD. Using EGFP-Met expression vector as a template, depletion mutants including KT EGFP-Met and kinase-only EGFP-Met were developed. Human full-length Met cDNA (with fused C-terminal HA tag) was subcloned into pcDNA3.1 Vector (Invitrogen, V79020). Human full-length Fis1 cDNA (with fused C-terminal Flag tag) was subcloned from FIS1 (Myc-DDK-tagged) (Origene, RC202560) and inserted into pcDNA3.1 Vector, PCDH-EF1-MCS-IRES Vector (System Biosciences, CD510B-1), or pGEX6P1 Vector (with fused N-terminal GST tag; GE Healthcare, GE28-9546-48). Using pcDNA3.1-Fis1-Flag as a template, several deletion mutants including Flag-Fis1-△α1, Flag-Fis1-△TPR1, Flag-Fis1-△TPR2, Flag-Fis1-(TPR2 + CT) were developed. Using PCDH-Fis1-Flag as a template, mutant plasmids including PCDH-Fis1-Flag-Y38E, PCDH-Fis1-Flag-Y38F, PCDH-Fis1-Flag-Y87E, PCDH-Fis1-Flag-Y87F, PCDH-Fis1-Flag-Y38/87E, PCDH-Fis1-Flag-Y38/87F were developed by performing site-directed mutagenesis. Human full-length Fis1 cDNA, Fis1-Y38F cDNA, Fis1-Y87F cDNA, Fis1-Y38/87F cDNA were subcloned into pGEX6P1 Vector with GST encoding sequence replaced by 6× His encoding sequence. Human full-length Fis1 cDNA, Fis1-Y38E cDNA, and Fis1-Y38F cDNA were subcloned into pECFP-C1 Vector (Clontech Laboratories, #6076-1). mCh-Drp1 (#49152) was obtained from addgene.^34^ Recombinant human HGF (CYT-244) was obtained from Prospec. Met inhibitors crizotinib (PF-02341066; S1068), tivantinib (ARQ-197; S2753), SU11274 (S1080), and mitochondrial fission inhibitor mdivi-1 (S7162) were purchased from Selleck Chemicals.

### Live cell imaging

HeLa cells expressing mCherry-Met with mitochondria stably marked by EGFP or Huh7 cells expressing WT or mutant EGFP-Met with mitochondria stably marked with mCherry were plated on 20-mm glass-bottomed dishes (NEST) at a density of 10,000 cells per dish. Images of cells were acquired using Olympus live cell imaging system using 100× oil objectives (for representative time-lapse images) at excitation wavelengths of 488 and 559 nm for EGFP and mCherry, respectively. CV1000 (YOKOGAWA) was used for imaging obtain. For mitochondrial fission events observation, images of cells were snapped at the interval of 15 s for 200 times with 7 slices ranging 3 µm.

### Confocal microscopy

All confocal images were acquired on Olympus FV1000 laser scanning confocal microscope with GaAsp detectors using a PlanApo λ 100 × 1.45 NA oil immersion objective (Olympus). Live cells were imaged in a temperature-controlled chamber (37 °C) at 5% CO_2_ at 1 frame every 2–3 s. Dual-color videos were acquired as consecutive green–red images and tricolor videos were acquired as consecutive green–red–blue images.

### Structured illumination microscopy

SIM super-resolution images were taken on a Nikon N-SIM system with a 100× oil immersion objective lens, 1.49 NA (Nikon). Images were captured using Nikon NIS-Elements and reconstructed using slice reconstruction in NIS elements. Images for live-cell imaging (live N-SIM) were taken at a single Z-plane, while images of fixed cells for 3D N-SIM were taken using Z-stacks with step sizes of 0.12 µm. Cells used for live-cell imaging were maintained in a temperature-controlled chamber (37 °C) at 5% CO_2_ in a TokaiHit stage top incubator.

### FRET imaging and analysis

HeLa cells were plated on 20-mm glass-bottomed dishes (NEST) at a density of 10,000 cells per dish. The following day, cells were transfected using lipofectamine with FRET pairs (mCh-DRP1 along with ECFP-Fis1 WT, ECFP-Fis1 Y38E, or EYFP-Fis1 Y38F). Cells were fixed in 4% (vol/vol) paraformaldehyde for 10 min and incubated in phosphate-buffered saline. Images of fixed HeLa cells were acquired using Olympus confocal microscope using 100× objectives (for representative time-lapse images) at excitation wavelengths of 453 nm, and 561 nm for ECFP and mCherry, respectively. FV1000 (Olympus) was used for FRET analysis to calculate SE-FRET and to unbiasedly generate regions of interest by tracing individual cells in the red fluorescence view. A total of *n* = 48 cells were analyzed per condition for Fis1 (WT), Fis1 (Y38E), and Fis1 (Y38F), and the FRET intensity was normalized to average SE-FRET values for Fis1 (WT).

### Image analysis

The mitochondrial skeleton structure was reconstructed using Imaris software. Mitochondrial localization of Met was defined as those Met dots overlapping with mitochondria over a quarter of its area. Mitochondrial contacts of Met imaged in living cells were categorized as those that showed mitochondria and Met dots in close proximity (<0.1 µm) for >10 s in time-lapse images. Mito-fission events were defined as those that showed the clear division of a single mitochondrion into two distinct daughter mitochondria that moved independently of one another after division.^35^ The expected probability that a Met dots would be at the site of a mito-fission event by random chance was calculated as the density of Met dots in the cytosol from *n* = 25 living cells, using ImageJ (National Institutes of Health (NIH)). All contacts analyzed for the duration were those that had already formed at the beginning of the video. The minimum duration of contact in HeLa cells was quantified as the time before contact termination and dissociation (Met dots and mitochondria detaching from one another) over a 5 min (300 s) video. Any contacts that lasted throughout the entire 5-min video and were still in contact by the end of the video were categorized as 300 s in bar graphs and as >5 min in histograms for the minimum duration of Met mitochondrial contacts. The percentage of Met in contacts with mitochondria was quantified as the percentage of vesicles that formed contacts (defined above) with mitochondria divided by the total number of vesicles in the region of interest. The duration of contact in cells was quantified from videos of ≥300 s. Mitochondrial morphology was analyzed using ImageJ (NIH), MiNA-Master system. The rate of mito-fission was defined by calculating the number of fission events per cell from videos of *n* = 25 living cells and ≥300 s.

### Statistical analysis

Unless otherwise noted, each experiment was repeated at least three times. All error bars represent the standard error of the mean. Student’s *t* test was used to compare two groups of independent samples. Repeated measure ANOVA analysis was used to evaluate the statistical significance of dose curve response. Correlations were analyzed using the Pearson chi-square test. A *p* value < 0.05 was considered statistically significant.

## Supplementary information

Supplementary Movie S1

Supplementary Movie S2

Supplementary Movie S3

Supplementary Movie S4

Supplementary Movie S5

Supplementary Movie S6

Supplementary Movie S7

Supplementary Materials

## Data Availability

All data supported the paper are presented in the paper and/or the Supplementary Materials. The original datasets are also available from the corresponding author upon request.
