# Supplementary material for: Fis1 phosphorylation by Met promotes mitochondrial fission and hepatocellular carcinoma metastasis
Source: Signal Transduct Target Ther. 2021 Dec 1;6:401. doi: 10.1038/s41392-021-00790-2 (PMC8632923; doi:10.1038/s41392-021-00790-2)
Supplement: Supplementary file 8 — Supplementary Materials [file 41392_2021_790_MOESM8_ESM.docx]

Supplementary Materials for

Fis1 Phosphorylation by Met Promotes Mitochondrial Fission and Hepatocellular Carcinoma Metastasis

Yan Yu^1#^, Xiao-Dan Peng^1#^, Xiao-Jun Qian^2#^, Kai-Ming Zhang^1#^,Xiang Huang^3^, Yu-Hong Chen^1^, Yun-Tian Li^2^, Gong-Kan Feng^1^, Hai-Liang Zhang^1^, Xue-Lian Xu^4^, Shun Li^1^, Xuan Li^1^, Jia Mai^1^, Zhi-Ling Li^1^, Yun Huang^1^, Dong Yang^1^, Li-Huan Zhou^1^, Zhuo-Yan Zhong^1^, Jun-Dong Li ^1,5^, Rong Deng^1*^, Xiao-Feng Zhu^1*^

Correspondence to: zhuxfeng@mail.sysu.edu.cn; dengrong@sysucc.org.cn

**This PDF file includes:**

Materials and Methods

Supplementary Figure Legends

Supplementary Figures S1 to S4

Supplementary Tables S1 to S3

Captions for Movies S1 to S7

**Other Supplementary Materials for this manuscript include the following:**

Movies S1 to S7

Materials and Methods

Cell culture and transfection

Human HEK293T, HeLa, and Huh7 cell lines were obtained from American Type Culture Collection (ATCC) and maintained in Dulbecco’s modified Eagle’s medium (DMEM; Gibco, C11995500BT) supplemented with 10% fetal bovine serum (FBS; Gibco, 10270-106) at 37 °C under 5% CO2. The cells were validated by short tandem repeat (STR) DNA fingerprinting using the AmpFLSTR® Identifiler® PCR Amplification Kit (Life Technologies, 4440211). The STR profiles were compared with ATCC fingerprints and the Cell Line Integrated Molecular Authentication database. For gene knockout, Fis1 CRISPR/Cas9 KO Plasmid (h) (Santa Cruz Biotechnology, sc-401007) or Met CRISPR/Cas9 KO Plasmid (h) (Santa Cruz Biotechnology, sc-400101-KO-2) were transfected into cells using Lipofectamine 2000 Reagent (Invitrogen, 11668019) with OptiMEM medium (Invitrogen, 31985070), positive cells with GFP fluorescence were dissociated by flow cytometry. For gene stable expression, indicated plasmids were co-transfected with packaging plasmids into HEK293T cells using branched polyethylenimine (PEI; Sigma-Aldrich, G04002) with complete DMEM, and viral particles were harvested 48 hours afterwards. Cells were infected with the viruses for 24 h with polybrene (8 μg/ml) and then selected by G418 for 7 days or puro for 3 days. For gene knockdown, siRNAs were transfected into cells using siRNA-Mate (Genepharma, G04002) according to the manufacturer’s protocol. The cells were collected for further experiment 48-72 hours after the transfection. Oligonucleotide sequences of siRNAs were listed as follows: MFF siRNA#1 (human): 5′-CCUUGUUCCAGGUCAGCGUUUGGCG-3′; MFF siRNA#2 (human): 5′-CGCCAAACGCUGACCUGGAACAAGG-3′. Knockout, knockdown or overexpression of the genes were validated by immunoblot.

Immunofluorescence

Cells were plated on coverslips and fixed in 4% (vol/vol) paraformaldehyde for 10 min and permeabilized with 1% BSA and 0.25% Triton-X-100. Fixed cells were incubated with primary antibodies for 1 h or overnight, washed three times for 5 min each time, incubated in secondary antibodies for 1 h, washed three times for 5 min each, and mounted on glass slides with ProLong™ Diamond Antifade Mountant (Invitrogen, P36970). To mark mitochondria, live cells were stained for 15 min at 37°C with 25 nM MitoTracker Green (Invitrogen, M7514) or 50 nM MitoTracker Red ((Invitrogen, M7512), and then subjected to fixation and permeabilization for subsequent manipulations described above.

Immunoprecipitation and immunoblotting

The sodium dodecyl sulfate-polyacrylamide gel electrophoresis (SDS-PAGE), Western blot and immunoprecipitation were performed as described previously^1^.

Mitochondrial isolation

Mitochondria of Huh7 cells were isolated through sucrose step-gradient centrifugation ^2,3^. After treatment by HGF (100 ng/ml) for 20min or treatment by crizotinib (1 μM), ARQ197 (5 μM), or SU11274 (1 μM) for 1 h, Huh7 cells were scraped down into ice-cold lysis buffer containing 10mM Tris-HCl of pH 7.8, 20% sucrose (v/v), 1% FBS, 0.5mM DTT and 1× protease inhibitor cocktail solution (Roche). Cells were homogenized in a tight-fitting Dounce homogenizer (Wheaton, Millville, NJ, USA) with 200 strokes. The homogenate was centrifuged at 850 g for 10 min at 4°C to remove the nuclei and cell debris. The supernatant was collected with clean EP tubes, and centrifuged at 3000 g for 15 min at 4°C. The supernatant was then collected as cytosolic fraction, and the pellet was twice washed as mitochondrial fraction.

In vitro kinase assay

Recombinant glutathione S-transferase (GST)-Fis1, His-Fis1 and mutants (His-Y38F, His-Y87F and His-Y38/87F) fusion proteins were expressed in BL12 E.coli and induced by isopropyl β-D-1-thiogalactopyranoside (IPTG) and purified with glutathione agarose beads. After cold-PBS washing 3 times, beads were suspended with 100 μl 1×tyrosine kinase buffer, with 25 μl saved for Western blotting. The beads were spun down and added with 100 μM ATP, 1 nM DTT, and 0.5 μg human recombinant active Met kinase, and incubated at 30 °C for 30 min. The kinase reaction was stopped by adding SDS loading dye and heating at 100 °C for 5 min. The samples were subjected to two identical SDS-PAGE. The One was applied to Coomassie blue staining of GST fusion Fis1 protein, and the other one was transferred and used to detect phosphorylation of substrate using Western blot.

Detection of mitochondrial activity and mitochondrial mass

Cells were washed in PBS and incubated for 20 min at 37℃ with MitoTracker Green (100 nM; Invitrogen, M7514) and tetramethylrhodamine ethyl ester perchlorate (TMRE; 100 nM; Sigma-Aldrich, 87917) in 0.2% BSA/PBS. After labeling, cells were washed two times with PBS and analyzed by FACS.

Measurement of apoptotic cells, ATP content and calcium influx

Apoptotic cells, ATP content and calcium influx were measured using Annexin V-FITC detection kit (Beyotime, C1062L), ATP Bioluminescence Assay Kit (Beyotime, S0026) and Fluo-4 AM calcium-ion fluorescent probe (Beyotime, S1060) respectively according to the manufacturer’s instructions.

Analysis of cell cycle

Cells were suspended in 100 µL PBS and fixed by 900 µL 70% (V/V) ethanol at 4 ℃ overnight. Centrifuge and wash the cells in cold PBS, and then suspend the cell pellet in Propidium Iodide (PI) staining solution (1 mg/mL PI and 20 mg/mL Rnase in PBS). Cell cycle was analyze by FACS.

Patient tissue samples and immunohistochemical staining

Human tumor tissue specimens were obtained from HCC patients with the first surgical resection at Sun Yet-sen University Cancer Center between 2012 and 2013 under the guidelines approved by the Ethics Committee. Patient tumor samples were deparaffinized and rehydrated. Antigen retrieval was carried out by heating in 0.01 M sodium-citrate buffer (pH 8.0) using a pressure-cooker. The sections were treated with 1% hydrogen peroxide in methanol for 30 min to block endogenous peroxidase activity. After 1 h preincubation in 10% fish skin protein to prevent nonspecific staining, the samples were incubated with primary antibodies at 4 °C overnight. The sections were then treated with biotinylated secondary antibody, followed by incubations with avidinbiotin peroxidase complex solution for 1 h at room temperature. Color was developed with the 3-amino-9-ethylcarbazole solution. Counterstaining was carried out using Mayer’s hematoxylin.

Mouse xenograft pulmonary metastasis models

All animal procedures were conducted under the approval of the Animal Ethics Committee at Sun Yet-sen University Cancer Center. Fis1 KO Huh7 cells rescued with Vector, Fis1 WT, Fis1 Y38E or Fis1 Y38F were injected to nude mice for 1×10^6^ cells /mouse through tail vein. Pulmonary metastasis was tested after 28 days (n = 8). Lungs were isolated after euthanasia and stained in picric acid for 6 h. And after fixation, lung tissues were embedded in paraffin, sectioned and stained with Hematoxylin and Eosin.

Mouse peritoneal metastasis models

All animal procedures were conducted under the approval of the Animal Ethics Committee at Sun Yet-sen University Cancer Center. Fis1 KO Huh7 cells rescued with Vector, Fis1 WT or Fis1 Y38F were intraperitoneally injected to nude mice (8 × 10^5^ cells /mouse). 21 days later, seeded nodes in the cavum abdominis were observed (n=5)^4^.

**Supplementary Figure Legends**

Figure. S1: Mitochondrial fission is driven by Met kinase.

a. Detection of ATP levels in Huh7 cells with HGF treatment (100 ng/ml, 20 min) or not. Error bars represent means ± SEM (n = 3 duplicate wells, NS denotes no statistical significance; Student’s t test).

b. Huh7 cells were treated with HGF (100 ng/ml, 20 min), the cytosolic Ca^2+^ was stained with Fluo-4AM and determined by flow cytometry. Error bars represent means ± SEM (NS denotes no statistical significance; Student’s t test). Data are representative of three independent experiments.

c. Huh7 cells were treated with HGF (100 ng/ml, 20 min), then apoptotic cells were stained with Annexin V/PI and analyzed by flow cytometry. The apoptotic cells including early apoptosis and late apoptosis were statistically presented. Error bars represent means ± SEM (NS denotes no statistical significance; Student’s t test). Data are representative of three independent experiments.

d. Huh7 cells were treated with HGF (100 ng/ml, 20 min) or not. Median Fluorescence Intensity (MFI) of TMRE and MTG were analyzed by flow cytometry. Error bars represent means ± SEM (NS denotes no statistical significance; Student’s t test). Data are representative of three independent experiments.

e. Immunoblot analysis of indicated proteins in mitochondrial and cytosolic fractions as well as total lysates of Huh7 cells treated with HGF (100 ng/ml, 20 min). Mitochondria isolation markers, α-tubulin (cytoplasmic) and Hsp60 (mitochondria) were used as controls.

f. Representative images of mitochondrial morphology in cells with HGF treatment (100 ng/ml, 20 min) or not, captured under Live Cell Imaging microscope. Scale bars, 10 µm.

g. Representative images of mitochondrial morphology in cells with crizotinib (1 µM, 1 h), ARQ197 (5 µM, 1 h) or SU11274 (1 µM, 1 h) or not, captured under Live Cell Imaging microscope. Scale bars, 10 µm.

Figure S2: Met interacts with Fis1 and triggers its tyrosine phosphorylation.

a. 293T cells with His-Met and Fis1-Flag overexpressed were stimulated with H_2_O_2_ (20 µM) for 15 min or not. Exogenous Fis1-Flag was purified with anti-Flag beads. Coomassie blue staining of proteins co-precipitating with Fis1-Flag was shown.

b. Proteomics analysis of proteins interacting with Fis1-Flag by Omicsbean, and the transmembrane receptor protein tyrosine kinase signaling pathway was shown, including Met.

c-e. HCC1806 (c), HT29 (d) and LO2 (e) cells were stimulated with HGF (100 ng/ml, 20 min) and crizotinib (1 µM, 1 h) or not and then applied to IP assay with anti-Fis1 antibody.

f. *Met^-/-^* Huh7 cells expressing exogenous Met were stimulated with HGF (100 ng/ml, 20 min) or not and then applied to IP assay with Flag beads. Immunoblot of indicated proteins was performed.

g. Quality of purified GST-Fis1 fusion protein controlled by BSA and kinase assay system related to Fig 4j were shown by Coomassie blue staining.

h. Quality of purified GST-Fis1 (WT), Fis1 (Y38F), Fis1 (Y87F) and Fis1 (Y38/87F) fusion proteins and kinase assay system related to Fig 4m were shown by Coomassie blue staining.

Figure S3: Y38 Phosphorylation of Fis1 facilitates Drp1 assembly to mitochondria and promotes mitochondrial fission.

a. Immunoblot analysis of Drp1 and Met in mitochondrial and cytosolic fractions of *Met^-/-^* Huh7 cells expressing Met WT or Met KD protein. Mitochondria isolation markers, GAPDH (cytoplasmic) and Hsp60 (mitochondria) were used as controls.

b. *Fis1^−/−^* Huh7 cells transfected with WT Fis1 or Y38F Fis1 were applied to IP assay with anti-Drp1 antibody. The interactions between Drp1 and indicated proteins were analyzed by western blot.

c. *Fis1^−/−^* Huh7 cells transfected with WT Fis1 or Y38F Fis1 were applied to IP assay with anti-Flag antibody. The interactions between Fis1 and indicated proteins were analyzed by western blot.

d. Representative images of Huh7 cells expressing empty vector, WT Fis1, Y38E Fis1, or Y38F Fis1, captured under Live Cell Imaging microscope. Scale bars, 10 µm.

Figure S4: Met mediated Fis1 Y38 phosphorylation facilitates cell metastasis *in vitro* and *in vivo*.

a. Representative images of wound healing assays in *Met^−/−^* Huh7 cells transfected with empty vector, WT Met WT, or KD Met, stimulated with HGF (100 ng/ml, 72 h) or not.

b. Representative images of transwell assay in *Met^−/−^* Huh7 cells implying the migration ability of individual clones. Quantification of cells migrated per field. Error bars represent means ± SEM (n = 8 fields, ***p* < 0.01; Student’s t test).

c. Representative images of extracellular matrix degradation assay in Huh7 cells with treatments of crizotinib (1 µM, 1 h), SU11274 (1 µM, 1 h) or ARQ-197 (5 µM, 1 h) testing the invasion ability.

d. Representative images of extracellular matrix degradation assay in *Met^−/−^* Huh7 cells testing the invasion ability.

e. Representative images of transwell assay in *Fis1^−/−^* Huh7 cells implying the migration ability of individual clones. Quantification of cells migrated per field. Error bars represent means ± SEM (n = 8 fields, ***p* < 0.01; Student’s t test).

f. Representative images of extracellular matrix degradation assay in WT or *Fis1^−/−^* Huh7 cells to test the invasion ability.

g. Representative images of transwell assay in cells with MFF silencing by siRNAs. Quantification of cells migrated per field. Error bars represent means ± SEM (n = 8 fields, NS denotes no statistical significance; Student’s t test).

h, i. WT or *Fis1^−/−^* Huh7 cells were injected to nude mice for 1×10^6^/ mouse through tail vein, pulmonary metastasis was tested after 28 days. Representative nodules on pulmonary surface after picric acid staining for 6 h **(h)** and images of lung histological sections with HE staining **(i, left)** were shown. Quantification of mean number of lung metastases per mice **(i, right)**. Error bars represent means ± SEM (n = 6 mice, ***p* < 0.01; Student’s t test).

j. Proliferation curves of *Fis1^−/−^* Huh7 cells expressing empty vector, WT Fis1, Y38E Fis1, or Y38F Fis1.

k. *Fis1^−/−^* Huh7 cells expressing empty vector, WT Fis1, Y38E Fis1, or Y38F Fis1 were stained with propidium iodide (PI) and cell cycle was determined by flow cytometry.

l. *Fis1^−/−^* Huh7 cells expressing empty vector, WT Fis1, Y38E Fis1, or Y38F Fis1 were treated with HGF (100 ng/ml, 20 min) or not, then apoptotic cells were stained with Annexin V/PI and analyzed by flow cytometry.

m. *Fis1^−/−^* Huh7 cells expressing empty vector, WT Fis1, Y38E Fis1, or Y38F Fis1 were treated with olaparib (left) or sorafenib (right) for 24 h and subjected to a cell viability assay.

n, o. Quantitative analysis of the migratory **(n)** and invasive **(l)** ability of Huh7 cells treated with crizotinib (1 µM), mdivi-1(10 µM) or the combination for 24 h. Representative images are shown (left). Quantification of migratory or invasive cells in each group (right). The migration or invasion of control cells (Vehicle) was set as 100%. Error bars represent means ± SEM ((**p* < 0.05, ***p* < 0.01; Student’s t test).


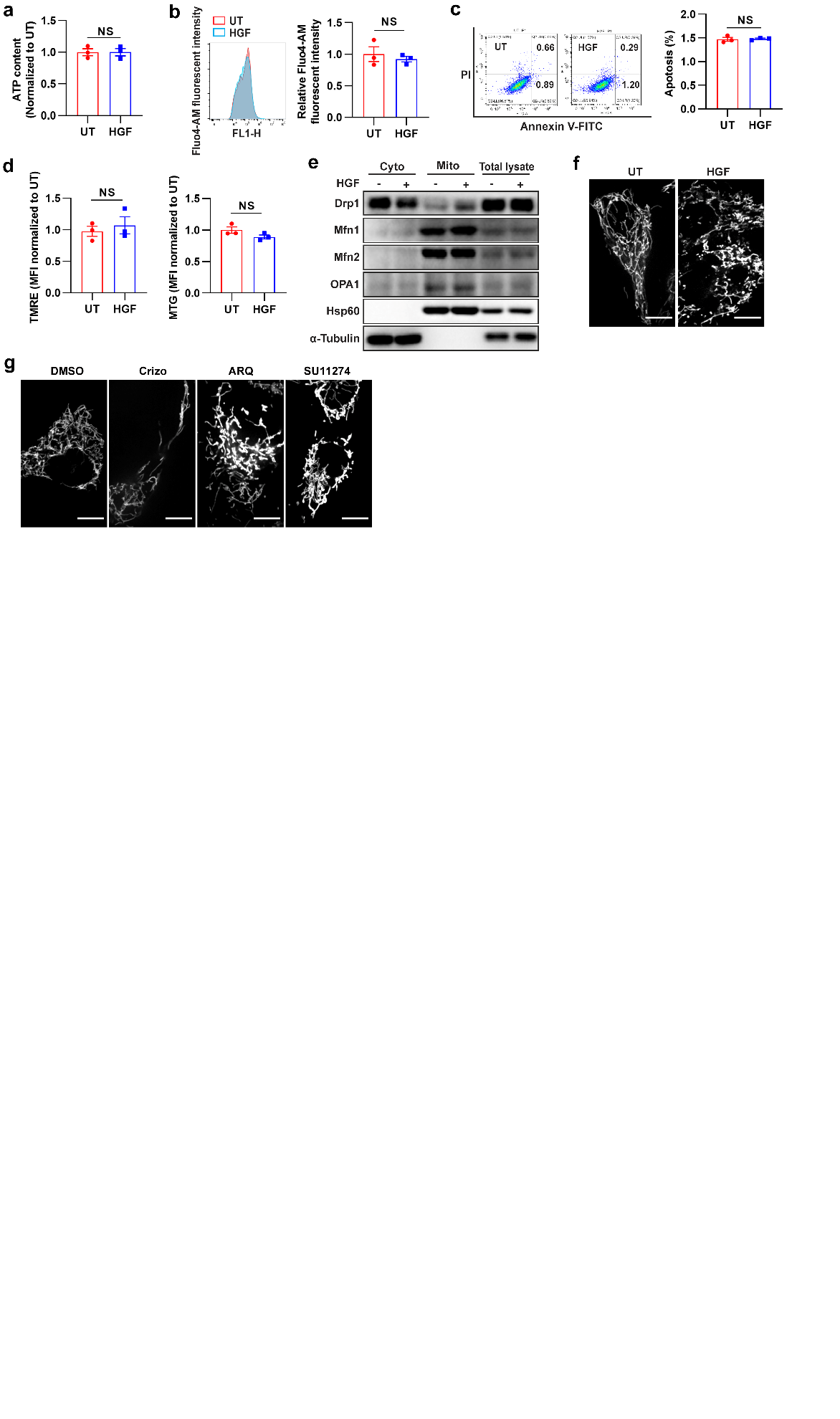


**Figure. S1.**

Mitochondrial fission is driven by Met kinase.


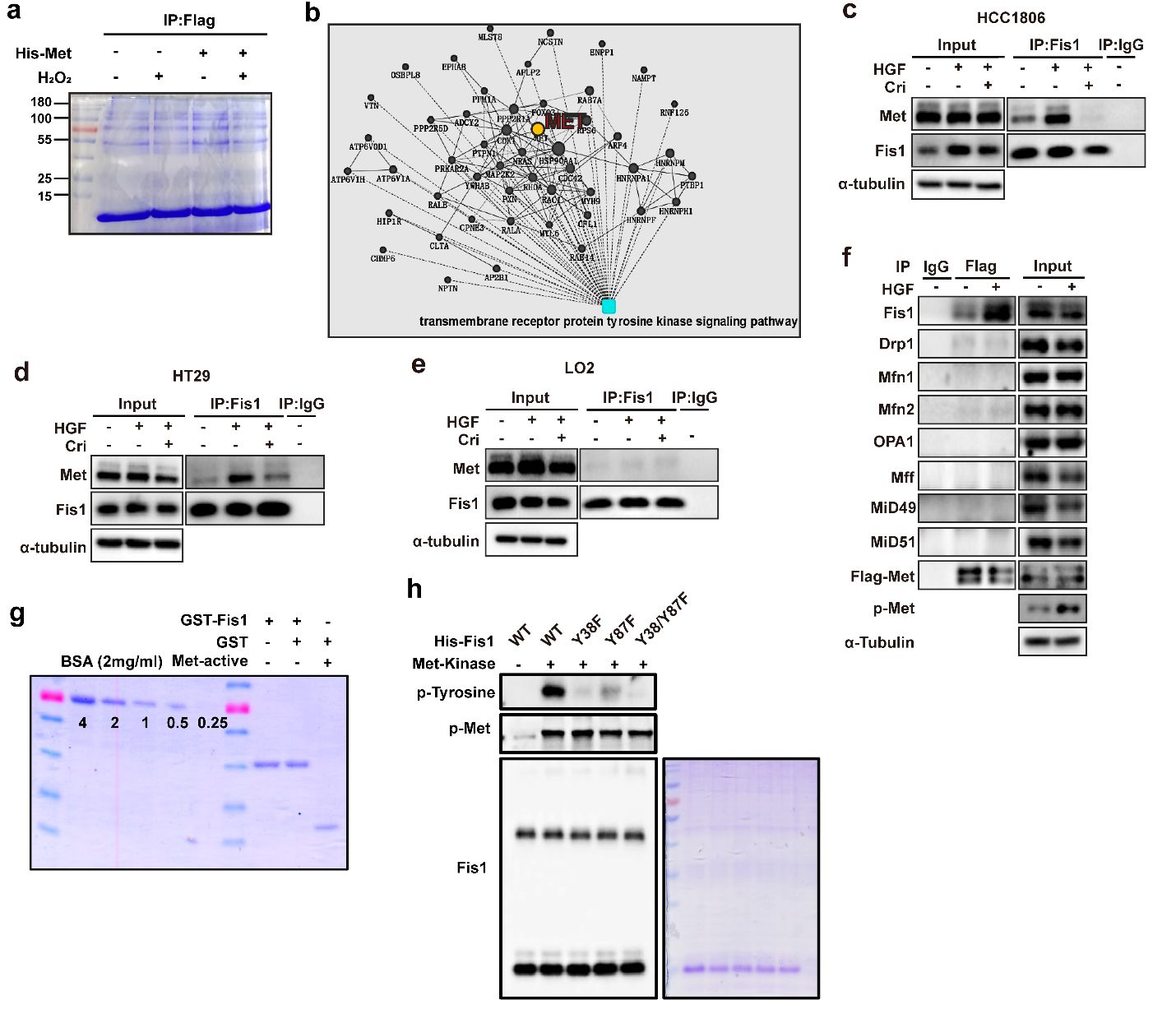


**Figure. S2.**

Met interacts with Fis1 and triggers its tyrosine phosphorylation.


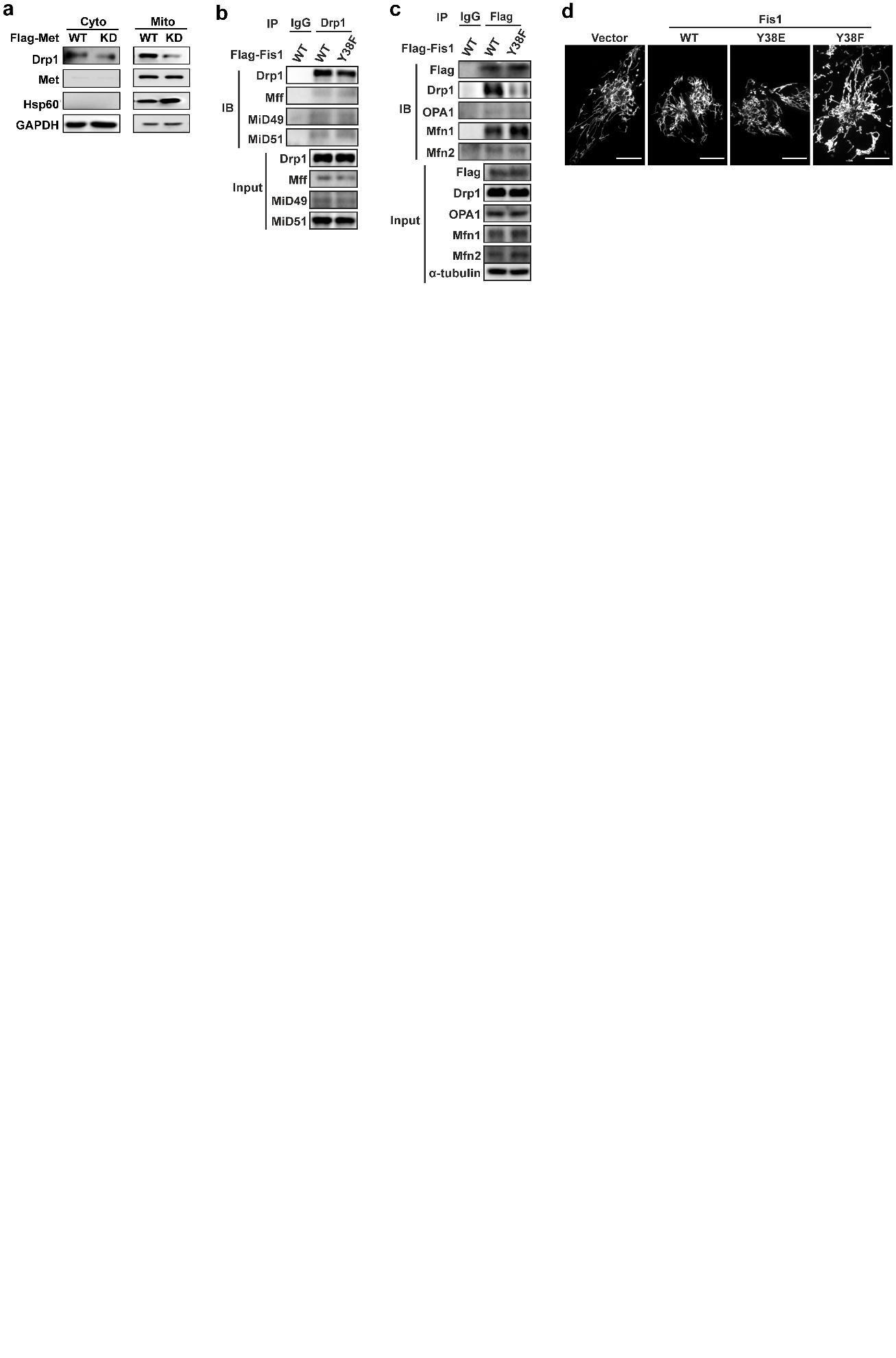


**Figure. S3.**

Y38 Phosphorylation of Fis1 facilitates Drp1 assembly to mitochondria and promotes mitochondrial fission.


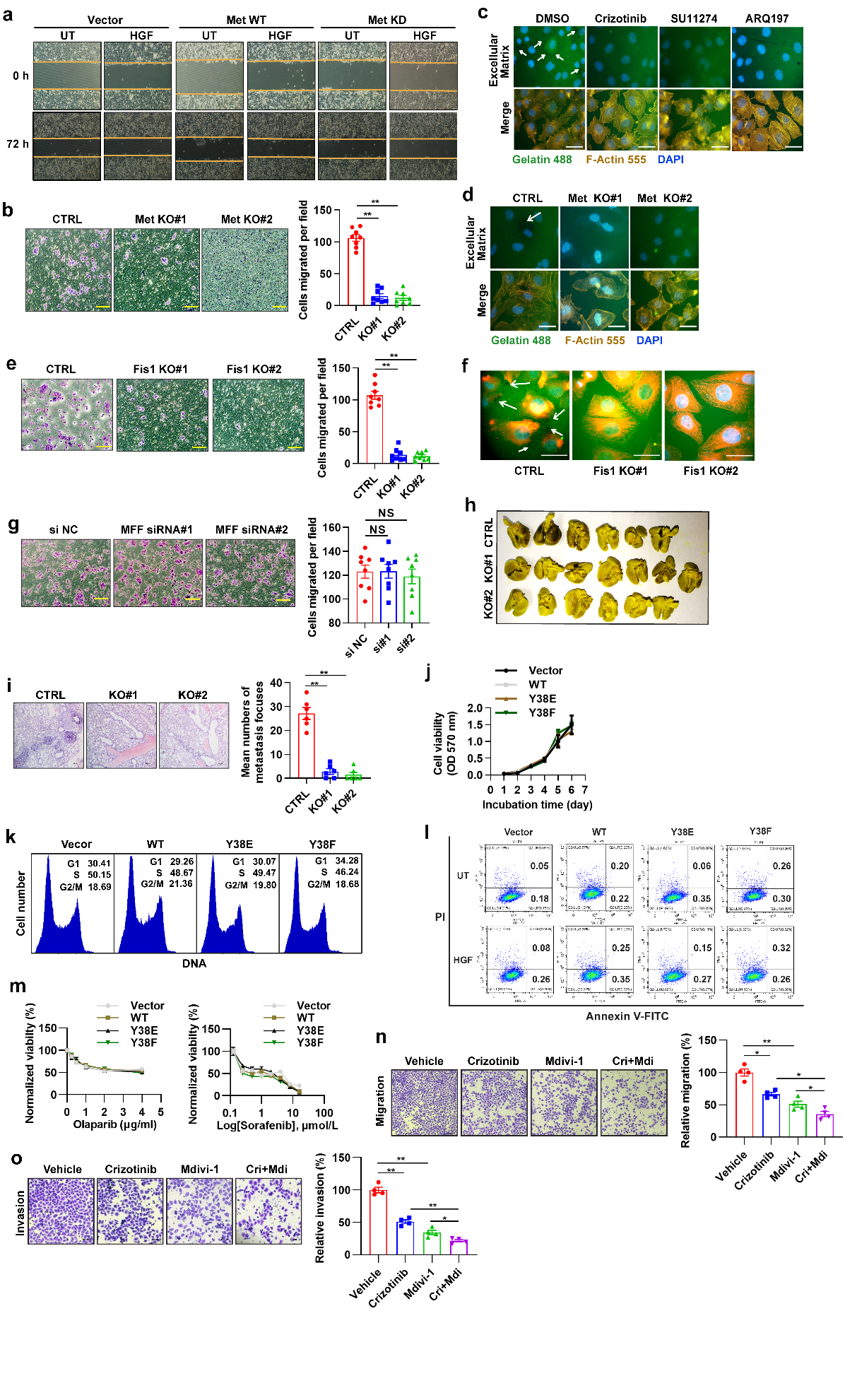


**Figure. S4.**

Met mediated Fis1 Y38 phosphorylation facilitates cell metastasis *in vitro* and *in vivo*.

**Table S1.** Clinicopathological data on 115 pairs of tumorous/non-tumorous HCC tissues.

| **Patient No*.** | **Gender (1=Male 0=Female)** | **Age at Diagnosis** | **Differenciation** | **Tumor Diameter (cm)** | **Paracancerous Distance (cm)** | **Over-all survival (OS) status (0=alive, 1=death)** | **OS time (Months)** | **Recurrence**  **(0=No, 1=Yes)** | **RFS time (Months)** |
| --- | --- | --- | --- | --- | --- | --- | --- | --- | --- |
| 626 | 1 | 49 | ModeratetoPoor | 4 | NA | 0 | 3.77 | 1 | 0.97 |
| 627 | 1 | 49 | other | 8 | NA | 0 | 37.17 | 1 | 6.97 |
| 629 | 1 | 62 | other | 6 | 3 | 0 | 50.37 | 0 | 50.37 |
| 630 | 0 | 67 | other | 2 | 2 | 0 | 33.87 | 1 | 19.47 |
| 631 | 0 | 46 | poor | 4 | 7 | 0 | 54.60 | 0 | 54.60 |
| 632 | 1 | 60 | poor | 1.2 | 2 | 0 | 0.43 | 0 | 0.43 |
| 633 | 1 | 54 | ModeratetoPoor | 9 | 3 | 0 | 10.77 | 0 | 10.77 |
| 634 | 1 | 66 | other | 10 | 4 | 0 | 49.43 | 1 | 35.53 |
| 635 | 0 | 30 | other | 7 | 6 | 0 | 49.90 | 0 | 49.90 |
| 639 | 1 | 54 | poor | 1.5 | 2 | 0 | 49.70 | 1 | 15.37 |
| 642 | 1 | 31 | ModeratetoPoor | 2.5 | 2 | 0 | 40.27 | 1 | 34.73 |
| 654 | 1 | 50 | other | 7 | 2 | 0 | 48.07 | 0 | 48.07 |
| 655 | 1 | 59 | ModeratetoPoor | 6 | NA | 1 | 13.93 | 1 | 2.07 |
| 656 | 1 | 62 | other | 3 | NA | 0 | 44.30 | 0 | 44.30 |
| 657 | 1 | 29 | ModeratetoPoor | 3 | 3 | 1 | 17.20 | 1 | 4.67 |
| 659 | 1 | 44 | other | 7 | 2.5 | 0 | 26.33 | 1 | 22.23 |
| 662 | 1 | 66 | other | 4.5 | 5 | 0 | 47.63 | 0 | 47.63 |
| 666 | 1 | 60 | other | 2 | 0.5 | 0 | 45.23 | 0 | 45.23 |
| 668 | 1 | 47 | other | 6 | 0.5 | 0 | 46.90 | 0 | 46.90 |
| 670 | 1 | 41 | other | 5 | 10 | 0 | 44.90 | 0 | 44.90 |
| 674 | 1 | 40 | other | 2.2 | 2 | 0 | 48.53 | 1 | 42.13 |
| 678 | 1 | 68 | other | 3 | 8 | 1 | 4.27 | 0 | 10.23 |
| 679 | 0 | 61 | other | 4 | 4 | 1 | 37.77 | 0 | 46.37 |
| 682 | 1 | 36 | other | 4 | 5 | 0 | 46.13 | 0 | 46.13 |
| 683 | 0 | 34 | other | 9 | 2 | 1 | 11.30 | 1 | 3.17 |
| 685 | 1 | 57 | other | 7 | 1.6 | 0 | 13.63 | 1 | 13.40 |
| 686 | 1 | 54 | poor | 7.2 | 4.5 | 0 | 49.47 | 0 | 49.47 |
| 687 | 1 | 47 | other | 5 | 4 | 0 | 1.37 | 0 | 1.37 |
| 688 | 1 | 50 | other | 9 | 0.5 | 0 | 34.13 | 0 | 34.13 |
| **Patient No*.** | **Gender (1=Male 0=Female)** | **Age at Diagnosis** | **Differenciation** | **Tumor Diameter (cm)** | **Paracancerous Distance (cm)** | **Over-all survival (OS) status (0=alive, 1=death)** | **OS time (Months)** | **Recurrence**  **(0=No, 1=Yes)** | **RFS time (Months)** |
| 689 | 1 | 54 | ModeratetoPoor | 7.5 | 1.5 | 1 | 31.00 | 1 | 1.13 |
| 691 | 0 | 39 | other | 3.2 | 2 | 0 | 47.80 | 0 | 47.80 |
| 692 | 0 | 82 | other | 5 | 2 | 0 | 45.47 | 0 | 45.47 |
| 693 | 1 | 47 | other | 2 | 1.5 | 0 | 45.37 | 0 | 45.37 |
| 695 | 1 | 42 | ModeratetoPoor | 1.5 | 6 | 0 | 48.90 | 1 | 42.57 |
| 696 | 1 | 60 | 0 | 4.8 | 2 | 0 | 84.60 | 1 | 50.77 |
| 697 | 1 | 44 | ModeratetoPoor | 5 | 0.5 | 0 | 10.20 | 1 | 4.17 |
| 698 | 1 | 57 | other | 7 | 3 | 0 | 20.27 | 1 | 11.80 |
| 701 | 0 | 55 | ModeratetoPoor | 3.5 | 1 | 0 | 48.10 | 0 | 48.10 |
| 705 | 1 | 66 | other | 1.5 | 1 | 1 | 34.80 | 1 | 12.27 |
| 707 | 1 | 44 | other | 7 | 1 | 0 | 48.33 | 0 | 48.33 |
| 708 | 1 | 56 | ModeratetoPoor | 5 | 3 | 0 | 48.23 | 0 | 48.23 |
| 709 | 1 | 34 | other | 1.5 | 2 | 0 | 48.03 | 1 | 5.47 |
| 710 | 1 | 61 | ModeratetoPoor | 6.5 | 2.5 | 0 | 44.10 | 0 | 44.10 |
| 711 | 0 | 62 | ModeratetoPoor | 4 | 1.5 | 0 | 44.10 | 0 | 44.10 |
| 712 | 1 | 43 | other | 9.5 | 2.5 | 0 | 43.87 | 1 | 7.23 |
| 713 | 1 | 60 | other | 2.5 | 1 | 0 | 47.87 | 1 | 21.90 |
| 720 | 1 | 61 | ModeratetoPoor | 6 | 2.5 | 1 | 11.77 | 1 | 2.50 |
| 723 | 1 | 39 | ModeratetoPoor | 11 | 3 | 1 | 7.40 | 0 | 12.33 |
| 726 | 1 | 59 | other | 4 | 3 | 1 | 10.23 | 1 | 3.03 |
| 727 | 1 | 28 | ModeratetoPoor | 15 | 3 | 0 | 45.87 | 1 | 14.83 |
| 729 | 1 | 58 | ModeratetoPoor | 2.5 | NA | 0 | 39.70 | 0 | 39.70 |
| 730 | 1 | 53 | poor | 1.5 | 3 | 1 | 26.80 | 0 | 34.03 |
| 731 | 1 | 55 | ModeratetoPoor | 4.5 | 4 | 0 | 43.10 | 0 | 43.10 |
| 733 | 1 | 40 | other | 3 | 3 | 0 | 41.43 | 0 | 41.43 |
| 734 | 0 | 39 | other | 8.5 | 3 | 0 | 1.13 | 0 | 1.13 |
| 735 | 1 | 54 | ModeratetoPoor | 6.5 | 2.5 | 0 | 0.27 | 0 | 0.27 |
| 739 | 1 | 63 | other | 3.5 | 4 | 0 | 38.90 | 0 | 38.90 |
| 740 | 1 | 53 | other | 8 | 4 | 0 | 40.87 | 0 | 40.87 |
| 742 | 1 | 50 | other | 3.5 | 2 | 0 | 41.00 | 0 | 41.00 |
| 743 | 1 | 39 | other | 7.5 | 4 | 0 | 1.77 | 0 | 1.77 |
| **Patient No*.** | **Gender (1=Male 0=Female)** | **Age at Diagnosis** | **Differenciation** | **Tumor Diameter (cm)** | **Paracancerous Distance (cm)** | **Over-all survival (OS) status (0=alive, 1=death)** | **OS time (Months)** | **Recurrence**  **(0=No, 1=Yes)** | **RFS time (Months)** |
| 745 | 1 | 43 | other | 3 | 3 | 0 | 44.53 | 0 | 44.53 |
| 747 | 1 | 49 | other | 4 | 1.5 | 0 | 42.53 | 1 | 30.53 |
| 748 | 1 | 38 | other | 1.5 | 2.5 | 0 | 38.03 | 0 | 38.03 |
| 749 | 1 | 47 | ModeratetoPoor | 8 | 0.5 | 1 | 10.07 | 1 | 3.87 |
| 750 | 1 | 42 | other | 3 | 1 | 0 | 38.20 | 0 | 38.20 |
| 751 | 1 | 42 | poor | 3 | 0.3 | 0 | 16.53 | 1 | 9.83 |
| 755 | 1 | 29 | other | 6 | 0.5 | 1 | 3.13 | 1 | 1.20 |
| 756 | 1 | 37 | poor | 10 | NA | 0 | 2.50 | 1 | 1.30 |
| 757 | 1 | 64 | other | 11 | 1.8 | 0 | 39.67 | 1 | 3.80 |
| 759 | 1 | 55 | other | 3.5 | 1.2 | 0 | 9.40 | 0 | 9.40 |
| 760 | 1 | 47 | other | 3 | 0.7 | 0 | 42.67 | 0 | 42.67 |
| 761 | 1 | 49 | other | 4.5 | 0.8 | 0 | 39.50 | 0 | 39.50 |
| 765 | 1 | 38 | other | 4 | 0.2 | 0 | 36.87 | 0 | 36.87 |
| 766 | 1 | 52 | other | 6 | 2.5 | 0 | 42.93 | 1 | 15.87 |
| 768 | 1 | 56 | ModeratetoPoor | 10 | 1 | 0 | 2.80 | 1 | 1.60 |
| 769 | 1 | 63 | other | 11 | 0.5 | 0 | 42.90 | 1 | 34.30 |
| 770 | 1 | 62 | other | 3 | 0.5 | 0 | 39.47 | 1 | 26.07 |
| 771 | 1 | 57 | other | 13 | 1.5 | 0 | 36.63 | 0 | 36.63 |
| 776 | 1 | 56 | other | 10 | Along the edge of the tumor | 0 | 35.97 | 0 | 35.97 |
| 777 | 1 | 76 | other | 5.5 | Along the edge of the tumor | 1 | 29.03 | 0 | 35.83 |
| 780 | 1 | 59 | poor | 1.5 | 2 | 0 | 38.77 | 1 | 36.07 |
| 781 | 1 | 45 | other | 5 | 1 | 0 | 41.40 | 0 | 41.40 |
| 788 | 0 | 40 | ModeratetoPoor | 3.5 | 2 | 0 | 36.10 | 0 | 36.10 |
| 789 | 1 | 45 | ModeratetoPoor | 5 | 2 | 1 | 27.13 | 1 | 5.00 |
| 790 | 1 | 63 | poor | 1.5 | 1.5 | 0 | 37.97 | 0 | 37.97 |
| 791 | 1 | 42 | other | 3.5 | 1 | 0 | 23.97 | 0 | 23.97 |
| 792 | 1 | 48 | other | 2.5 | 0.5 | 0 | 35.47 | 1 | 32.20 |
| 793 | 1 | 36 | ModeratetoPoor | 13 | 0.3 | 0 | 1.23 | 1 | 1.20 |
| 794 | 1 | 59 | other | 1.5 | 1.3 | 0 | 38.90 | 1 | 25.30 |
| **Patient No*.** | **Gender (1=Male 0=Female)** | **Age at Diagnosis** | **Differenciation** | **Tumor Diameter (cm)** | **Paracancerous Distance (cm)** | **Over-all survival (OS) status (0=alive, 1=death)** | **OS time (Months)** | **Recurrence**  **(0=No, 1=Yes)** | **RFS time (Months)** |
| 795 | 1 | 70 | other | 3.5 | 0.5 | 1 | 16.93 | 1 | 2.43 |
| 797 | 1 | 46 | ModeratetoPoor | 3.5 | 0.2 | 0 | 39.70 | 0 | 39.70 |
| 798 | 1 | 55 | ModeratetoPoor | 3.5 | 1 | 1 | 12.07 | 1 | 5.80 |
| 799 | 1 | 56 | ModeratetoPoor | 5 | 0 | 0 | 33.17 | 0 | 33.17 |
| 800 | 1 | 36 | ModeratetoPoor | 8 | 1.3 | 0 | 38.10 | 0 | 38.10 |
| 801 | 1 | 63 | other | 2 | 0.2 | 0 | 37.17 | 0 | 37.17 |
| 802 | 1 | 49 | poor | 12 | 0.7 | 0 | 37.47 | 0 | 37.47 |
| 803 | 0 | 54 | other | 2.5 | 1.5 | 0 | 37.60 | 1 | 3.93 |
| 804 | 1 | 73 | other | 5.5 | 0.2; 0.6 | 0 | 29.53 | 1 | 29.07 |
| 805 | 1 | 43 | other | 2 | 0.8 | 0 | 37.73 | 1 | 26.57 |
| 806 | 0 | 65 | poor | 2 | 1 | 0 | 37.80 | 1 | 14.90 |
| 807 | 1 | 54 | ModeratetoPoor | 2.8 | 0.3 | 0 | 36.53 | 0 | 36.53 |
| 809 | 1 | 61 | other | 3.5 | 1.5 | 0 | 38.27 | 0 | 38.27 |
| 810 | 1 | 31 | other | 4 | 0.8 | 0 | 37.57 | 1 | 16.63 |
| 811 | 0 | 47 | other | 5.3 | 0.7 | 0 | 36.00 | 1 | 7.83 |
| 818 | 1 | 34 | other | 6 | 0.9 | 0 | 34.33 | 1 | 1.13 |
| 819 | 1 | 44 | poor | 6.5 | 0.3 | 0 | 11.23 | 1 | 9.33 |
| 820 | 1 | 41 | ModeratetoPoor | 8 | 0.5\3 | 0 | 28.87 | 1 | 1.00 |
| 821 | 1 | 59 | ModeratetoPoor | 7 | 0.7 | 0 | 31.43 | 1 | 15.77 |
| 822 | 1 | 54 | other | 4 | 1.3 | 0 | 31.43 | 1 | 13.73 |
| 823 | 1 | 50 | other | 12.5 | 0 | 0 | 38.07 | 0 | 38.07 |
| 825 | 1 | 37 | other | 0.8 | 3 | 0 | 11.43 | 0 | 11.43 |
| 826 | 1 | 56 | other | 4.5 | 1.4 | 0 | 37.17 | 0 | 37.17 |
| 829 | 1 | 50 | other | 2 | 0.3 | 0 | 36.20 | 1 | 11.40 |
| 830 | 1 | 59 | other | 4 | 0 | 0 | 35.10 | 0 | 35.10 |
| 831 | 1 | 38 | ModeratetoPoor | 4.5 | 0 | 0 | 36.73 | 0 | 36.73 |

**Table S2.** Clinicopathological data on 177 cases included in the HCC tissue microarray and HGF/p-Fis1/p-Met expression in these samples measured by immunohistochemical staining.

| **Patient No*.** | **Gender (1=Male 0=Female)** | **Age at Diagnosis** | **HGF score** | **HGF expression** | **p-Fis1 score** | | **p-Fis1 expression** | **p-Met score** | **p-Met expression** | **Over-all survival (OS) status (0=alive, 1=death)** | **OS time (Months)** |
| --- | --- | --- | --- | --- | --- | --- | --- | --- | --- | --- | --- |
| 641 | 1 | 35 | 0 | Low | | 0 | Low | 6 | High | 0 | 114 |
| 647 | 1 | 56 | 1.5 | High | | 2 | High | 6 | High | 1 | 13 |
| 648 | 1 | 20 | 2 | High | | 2 | High | 6 | High | 1 | 17 |
| 654 | 0 | 33 | 1.5 | High | | 1.5 | Low | 4 | Low | 0 | 107 |
| 657 | 1 | 40 | 2 | High | | 2 | High | 4 | Low | 1 | 3 |
| 662 | 1 | 58 | 2 | High | | 2 | High | 9 | High | 1 | 16 |
| 663 | 1 | 59 | 1 | Low | | 1.5 | Low | 6 | High | 0 | 18 |
| 666 | 1 | 56 | 1.5 | High | | 1 | Low | 6 | High | 0 | 116 |
| 690 | 1 | 51 | 1 | Low | | 1 | Low | 9 | High | 1 | 3 |
| 693 | 1 | 73 | 0 | Low | | 0 | Low | 1 | Low | 1 | 4 |
| 707 | 1 | 59 | 0.5 | Low | | 1 | Low | 6 | High | 1 | 59 |
| 708 | 1 | 65 | 1.5 | High | | 1.5 | Low | 6 | High | 1 | 11 |
| 720 | 1 | 35 | 1 | Low | | 1.5 | Low | 4 | Low | 1 | 11 |
| 729 | 0 | 49 | 3 | High | | 3 | High | 6 | High | 1 | 3 |
| 731 | 1 | 39 | 3 | High | | 3 | High | 3 | Low | 1 | 6 |
| 732 | 0 | 54 | 0 | Low | | 0 | Low | 2 | Low | 1 | 20 |
| 734 | 1 | 59 | 0 | Low | | 0 | Low | 5 | Low | 0 | 118 |
| 735 | 1 | 32 | 0.5 | Low | | 0.5 | Low | 4 | Low | 0 | 115 |
| 736 | 1 | 33 | 0 | Low | | 0 | Low | 2 | Low | 0 | 114 |
| 737 | 1 | 40 | 2 | High | | 2 | High | 12 | High | 1 | 3 |
| 738 | 1 | 56 | 0.5 | Low | | 1 | Low | 4 | Low | 0 | 103 |
| 739 | 1 | 59 | 0 | Low | | 1 | Low | 8 | High | 1 | 7 |
| 741 | 1 | 36 | 3 | High | | 2.5 | High | NA |  | 1 | 83 |
| 742 | 1 | 50 | 2.5 | High | | 2.5 | High | 6 | High | 1 | 5 |
| 744 | 1 | 48 | 1 | Low | | 1 | Low | 5 | Low | 0 | 82 |
| 745 | 1 | 49 | 1 | Low | | 1 | Low | NA |  | 0 | 82 |
| **Patient No*.** | **Gender (1=Male 0=Female)** | **Age at Diagnosis** | **HGF score** | **HGF expression** | **p-Fis1 score** | | **p-Fis1 expression** | **p-Met score** | **p-Met expression** | **Over-all survival (OS) status (0=alive, 1=death)** | **OS time (Months)** |
| 746 | 1 | 59 | 1 | Low | | 1 | Low | 7 | High | 0 | 114 |
| 749 | 1 | 42 | 1 | Low | | 1 | Low | 5 | Low | 0 | 101 |
| 767 | 1 | 33 | 3 | High | | 3 | High | 6 | High | 1 | 17 |
| 783 | 0 | 46 | 2 | High | | 2 | High | 6 | High | 1 | 9 |
| 784 | 1 | 36 | 3 | High | | 3 | High | 6 | High | 1 | 6 |
| 785 | 1 | 36 | 1 | Low | | 1 | Low | 7 | High | 0 | 130 |
| 786 | 1 | 51 | 1 | Low | | 1 | Low | 6 | High | 0 | 114 |
| 788 | 1 | 53 | 2 | High | | 2 | High | 12 | High | 1 | 58 |
| 792 | 1 | 33 | 3 | High | | 3 | High | 8 | High | 1 | 2 |
| 793 | 1 | 45 | 3 | High | | 3 | High | 4 | Low | 1 | 7 |
| 798 | 1 | 38 | 3 | High | | 3 | High | 6 | High | 1 | 13 |
| 822 | 1 | 38 | 1 | Low | | 1 | Low | 4 | Low | 0 | 127 |
| 828 | 1 | 69 | 2 | High | | 1.5 | Low | 8.5 | High | 0 | 36 |
| 830 | 1 | 64 | 3 | High | | 3 | High | 4 | Low | 1 | 7 |
| 831 | 0 | 65 | 2.5 | High | | 2.5 | High | 4 | Low | 1 | 9 |
| 834 | 1 | 43 | 0.5 | Low | | 0.5 | Low | 5 | Low | 0 | 112 |
| 835 | 1 | 65 | 3 | High | | 2 | High | 7 | High | 1 | 62 |
| 836 | 0 | 49 | 1 | Low | | 1 | Low | 4 | Low | 0 | 116 |
| 838 | 1 | 50 | 1.5 | High | | 1.5 | Low | 5 | Low | 0 | 126 |
| 841 | 1 | 31 | 1 | Low | | 1 | Low | 4 | Low | 0 | 125 |
| 846 | 0 | 41 | 2 | High | | 2 | High | 9 | High | 1 | 10 |
| 847 | 1 | 19 | 1.5 | High | | 2 | High | 6 | High | 0 | 45 |
| 848 | 1 | 60 | 0.5 | Low | | 0.5 | Low | 4 | Low | 0 | 107 |
| 855 | 1 | 67 | 2.5 | High | | 2.5 | High | 6 | High | 1 | 17 |
| 864 | 0 | 68 | 2 | High | | 2 | High | 5 | Low | 0 | 114 |
| 876 | 1 | 44 | 2.5 | High | | 2.5 | High | 6 | High | 1 | 4 |
| 879 | 1 | 61 | 2 | High | | 2 | High | 6 | High | 0 | 86 |
| 880 | 1 | 55 | 3 | High | | 3 | High | 6 | High | 1 | 4 |
| 882 | 0 | 26 | 0.5 | Low | | 0.5 | Low | 4 | Low | 0 | 100 |
| **Patient No*.** | **Gender (1=Male 0=Female)** | **Age at Diagnosis** | **HGF score** | **HGF expression** | **p-Fis1 score** | | **p-Fis1 expression** | **p-Met score** | **p-Met expression** | **Over-all survival (OS) status (0=alive, 1=death)** | **OS time (Months)** |
| 883 | 1 | 34 | 0 | Low | | 0 | Low | 6 | High | 0 | 99 |
| 884 | 1 | 60 | 3 | High | | 3 | High | 6 | High | 1 | 6 |
| 896 | 1 | 46 | 3 | High | | 3 | High | 6 | High | 1 | 10 |
| 897 | 1 | 35 | 0 | Low | | 0.5 | Low | 8 | High | 0 | 103 |
| 900 | 1 | 47 | 0.5 | Low | | 1 | Low | 6 | High | 1 | 65 |
| 901 | 1 | 35 | 0 | Low | | 0 | Low | 4 | Low | 0 | 90 |
| 909 | 1 | 44 | 0 | Low | | 0 | Low | 4 | Low | 0 | 111 |
| 915 | 1 | 26 | 2 | High | | 3 | High | 4 | Low | 1 | 4 |
| 916 | 1 | 49 | 2 | High | | 3 | High | 3 | Low | 1 | 8 |
| 924 | 1 | 71 | 0 | Low | | 0.5 | Low | 4 | Low | 0 | 120 |
| 957 | 1 | 58 | 0 | Low | | 0 | Low | 5 | Low | 0 | 109 |
| 1067 | 1 | 53 | 0 | Low | | 1 | Low | 4 | Low | 0 | 22 |
| 1071 | 1 | 54 | 3 | High | | 3 | High | 6 | High | 1 | 7 |
| 1072 | 1 | 73 | 1.5 | High | | 2.5 | High | 6 | High | 0 | 104 |
| 1073 | 1 | 59 | 2 | High | | 2 | High | 4 | Low | 1 | 11 |
| 1075 | 1 | 41 | 0 | Low | | 1 | Low | 4 | Low | 0 | 112 |
| 1078 | 1 | 66 | 2 | High | | 2 | High | 6 | High | 1 | 16 |
| 1079 | 1 | 69 | 1 | Low | | 1 | Low | 6 | High | 1 | 65 |
| 1081 | 1 | 58 | 1 | Low | | 1 | Low | 6 | High | 0 | 72 |
| 1108 | 1 | 58 | 2 | High | | 2 | High | 6 | High | 1 | 7 |
| 1126 | 1 | 57 | 0.5 | Low | | 1 | Low | 12 | High | 0 | 97 |
| 1171 | 1 | 46 | 0 | Low | | 1 | Low | 4 | Low | 0 | 40 |
| 1196 | 1 | 44 | 0 | Low | | 0 | Low | 2 | Low | 0 | 87 |
| 1200 | 1 | 53 | 2.5 | High | | 2 | High | 4 | Low | 1 | 28 |
| 1206 | 1 | 40 | 1 | Low | | 1 | Low | 9 | High | 0 | 103 |
| 1208 | 1 | 39 | 1.5 | High | | 1.5 | Low | 6 | High | 0 | 88 |
| 1213 | 1 | 41 | 0 | Low | | 0.5 | Low | 8 | High | 0 | 104 |
| 1217 | 1 | 64 | 2 | High | | 2.5 | High | 6 | High | 1 | 8 |
| 1223 | 1 | 45 | 0.5 | Low | | 0 | Low | 6 | High | 0 | 94 |
| **Patient No*.** | **Gender (1=Male 0=Female)** | **Age at Diagnosis** | **HGF score** | **HGF expression** | **p-Fis1 score** | | **p-Fis1 expression** | **p-Met score** | **p-Met expression** | **Over-all survival (OS) status (0=alive, 1=death)** | **OS time (Months)** |
| 1225 | 1 | 46 | 0.5 | Low | | 1 | Low | 9 | High | 0 | 103 |
| 1237 | 1 | 54 | 2 | High | | 1 | Low | 6 | High | 0 | 103 |
| 1279 | 1 | 23 | 1.5 | High | | 2 | High | 12 | High | 0 | 36 |
| 1319 | 1 | 42 | 1 | Low | | 0 | Low | 6 | High | 0 | 98 |
| 1340 | 1 | 42 | 0 | Low | | 0 | Low | 7.5 | High | 0 | 55 |
| 1382 | 1 | 35 | 1 | Low | | 2 | High | 9 | High | 1 | 12 |
| 1388 | 1 | 39 | 0.5 | Low | | 0.5 | Low | 9 | High | 0 | 95 |
| 1401 | 1 | 56 | 1.5 | High | | 1.5 | Low | 6 | High | 0 | 93 |
| 1409 | 1 | 58 | 2 | High | | 2 | High | 12 | High | 1 | 10 |
| 1455 | 1 | 58 | 1 | Low | | 1 | Low | 4 | Low | 0 | 82 |
| 1485 | 1 | 58 | 1 | Low | | 1 | Low | 6 | High | 0 | 69 |
| 1487 | 1 | 53 | 2 | High | | 2 | High | 2 | Low | 1 | 8 |
| 1489 | 1 | 36 | 1 | Low | | 1.5 | Low | 6 | High | 1 | 17 |
| 1493 | 1 | 60 | 3 | High | | 3 | High | 9 | High | 1 | 12 |
| 1508 | 1 | 57 | 2 | High | | 3 | High | 6 | High | 1 | 11 |
| 1519 | 1 | 65 | 0 | Low | | 0 | Low | 6 | High | 0 | 87 |
| 1522 | 1 | 55 | 0 | Low | | 1 | Low | 6 | High | 0 | 88 |
| 1529 | 1 | 51 | 2 | High | | 3 | High | 4 | Low | 1 | 23 |
| 1532 | 1 | 52 | 0.5 | Low | | 0 | Low | 3 | Low | 0 | 74 |
| 1536 | 1 | 31 | 2.5 | High | | 2 | High | 2 | Low | 1 | 4 |
| 1538 | 1 | 48 | 0 | Low | | 0 | Low | 6 | High | 0 | 85 |
| 1545 | 1 | 48 | 1.5 | High | | 2.5 | High | 3 | Low | 1 | 2 |
| 1549 | 1 | 38 | 0 | Low | | 0 | Low | 6 | High | 0 | 86 |
| 1558 | 0 | 53 | 2 | High | | 1.5 | Low | 2 | Low | 1 | 24 |
| 1564 | 1 | 34 | 1 | Low | | 1 | Low | 1 | Low | 1 | 9 |
| 1577 | 1 | 66 | 2.5 | High | | 3 | High | 6 | High | 1 | 38 |
| 1578 | 1 | 60 | 0.5 | Low | | 0.5 | Low | 9 | High | 0 | 81 |
| 1583 | 1 | 42 | 2 | High | | 2.5 | High | 2 | Low | 1 | 10 |
| 1587 | 1 | 54 | 2 | High | | 2.5 | High | 4 | Low | 1 | 30 |
| **Patient No*.** | **Gender (1=Male 0=Female)** | **Age at Diagnosis** | **HGF score** | **HGF expression** | **p-Fis1 score** | | **p-Fis1 expression** | **p-Met score** | **p-Met expression** | **Over-all survival (OS) status (0=alive, 1=death)** | **OS time (Months)** |
| 1589 | 1 | 51 | 0.5 | Low | | 0.5 | Low | 4 | Low | 0 | 83 |
| 1592 | 1 | 46 | 3 | High | | 2 | High | 6 | High | 1 | 4 |
| 1593 | 1 | 61 | 0 | Low | | 0 | Low | 6 | High | 0 | 69 |
| 1595 | 1 | 65 | 2 | High | | 3 | High | 6 | High | 1 | 12 |
| 1602 | 1 | 58 | 0.5 | Low | | 1 | Low | 6 | High | 0 | 77 |
| 1603 | 1 | 59 | 2 | High | | 1.5 | Low | 2 | Low | 1 | 18 |
| 1606 | 1 | 52 | 1 | Low | | 1 | Low | 4 | Low | 0 | 84 |
| 1615 | 1 | 58 | 1.5 | High | | 2 | High | 8 | High | 1 | 11 |
| 1617 | 0 | 59 | 1 | Low | | 1 | Low | 4 | Low | 0 | 82 |
| 1618 | 1 | 47 | 2 | High | | 2 | High | 6 | High | 1 | 40 |
| 1619 | 1 | 40 | 1.5 | High | | 1.5 | Low | 4 | Low | 0 | 16 |
| 1621 | 1 | 48 | 1.5 | High | | 1 | Low | 4 | Low | 0 | 47 |
| 1622 | 1 | 43 | 2 | High | | 2 | High | 3 | Low | 1 | 32 |
| 1626 | 1 | 43 | 0.5 | Low | | 1 | Low | 4 | Low | 0 | 84 |
| 1629 | 1 | 42 | 0.5 | Low | | 1 | Low | 2 | Low | 0 | 60 |
| 1631 | 1 | 52 | 2 | High | | 2 | High | 6 | High | 1 | 10 |
| 1635 | 1 | 60 | 2.5 | High | | 3 | High | 6 | High | 1 | 12 |
| 1636 | 1 | 41 | 0.5 | Low | | 0.5 | Low | 9 | High | 0 | 83 |
| 1641 | 0 | 58 | 0.5 | Low | | 1 | Low | 6 | High | 0 | 79 |
| 1643 | 1 | 54 | 1 | Low | | 2 | High | 3 | Low | 0 | 79 |
| 1649 | 1 | 54 | 2 | High | | 2 | High | 3 | Low | 1 | 28 |
| 1650 | 1 | 46 | 0 | Low | | 0 | Low | 1 | Low | 0 | 81 |
| 1658 | 1 | 41 | 1 | Low | | 0.5 | Low | 6 | High | 0 | 65 |
| 1661 | 0 | 57 | 2 | High | | 2 | High | 6 | High | 1 | 14 |
| 1668 | 1 | 59 | 1 | Low | | 1 | Low | 6 | High | 0 | 80 |
| 1669 | 1 | 37 | 2.5 | High | | 2.5 | High | 6 | High | 1 | 7 |
| 1670 | 1 | 53 | 1 | Low | | 0 | Low | 3 | Low | 0 | 80 |
| 1671 | 1 | 50 | 0 | Low | | 0 | Low | 2 | Low | 0 | 28 |
| 1672 | 1 | 39 | 0.5 | Low | | 1 | Low | 9 | High | 0 | 80 |
| **Patient No*.** | **Gender (1=Male 0=Female)** | **Age at Diagnosis** | **HGF score** | **HGF expression** | **p-Fis1 score** | | **p-Fis1 expression** | **p-Met score** | **p-Met expression** | **Over-all survival (OS) status (0=alive, 1=death)** | **OS time (Months)** |
| 1676 | 1 | 37 | 2 | High | | 2 | High | 8 | High | 1 | 12 |
| 1678 | 1 | 48 | 2.5 | High | | 2.5 | High | 6 | High | 1 | 8 |
| 1681 | 1 | 49 | 2 | High | | 1.5 | Low | 8 | High | 0 | 82 |
| 1683 | 1 | 38 | 1.5 | High | | 2 | High | 6 | High | 1 | 6 |
| 1685 | 1 | 45 | 0 | Low | | 1 | Low | 8 | High | 0 | 72 |
| 1686 | 1 | 46 | 0 | Low | | 0.5 | Low | 3 | Low | 0 | 81 |
| 1689 | 1 | 54 | 2.5 | High | | 2.5 | High | 6 | High | 1 | 16 |
| 1692 | 1 | 45 | 1 | Low | | 0 | Low | 8 | High | 0 | 82 |
| 1693 | 1 | 52 | 2.5 | High | | 2 | High | 6 | High | 1 | 23 |
| 1699 | 1 | 33 | 1 | Low | | 0.5 | Low | 4 | Low | 0 | 77 |
| 1711 | 1 | 38 | 1 | Low | | 0 | Low | 4 | Low | 0 | 69 |
| 1713 | 1 | 36 | 2.5 | High | | 2 | High | 3 | Low | 1 | 11 |
| 1714 | 1 | 26 | 0.5 | Low | | 1 | Low | 3 | Low | 0 | 71 |
| 1718 | 1 | 58 | 2.5 | High | | 2 | High | 6 | High | 1 | 43 |
| 1729 | 1 | 54 | 2.5 | High | | 3 | High | 9 | High | 1 | 17 |
| 1751 | 1 | 50 | 3 | High | | 3 | High | 8 | High | 1 | 5 |
| 1753 | 1 | 37 | 1 | Low | | 1 | Low | 2 | Low | 0 | 78 |
| 1764 | 1 | 43 | 2.5 | High | | 2.5 | High | 6 | High | 1 | 50 |
| 1768 | 1 | 44 | 0 | Low | | 0.5 | Low | 2 | Low | 0 | 76 |
| 1773 | 1 | 56 | 0 | Low | | 0.5 | Low | 2 | Low | 0 | 69 |
| 1776 | 0 | 39 | 2 | High | | 2 | High | 3 | Low | 1 | 20 |
| 1778 | 0 | 65 | 3 | High | | 3 | High | 4 | Low | 1 | 39 |
| 1790 | 0 | 49 | 0.5 | Low | | 0 | Low | 6 | High | 0 | 72 |
| 1795 | 1 | 48 | 0.5 | Low | | 0 | Low | 9 | High | 0 | 71 |
| 1823 | 1 | 57 | 0.5 | Low | | 0.5 | Low | 3 | Low | 0 | 75 |
| 1828 | 1 | 36 | 0 | Low | | 1 | Low | 9 | High | 0 | 75 |
| 1874 | 1 | 57 | 0.5 | Low | | 1 | Low | NA |  | 0 | 68 |
| 1894 | 1 | 48 | 1 | Low | | 0.5 | Low | 4 | Low | 0 | 47 |
| 1903 | 0 | 57 | 0.5 | Low | | 0 | Low | 4 | Low | 0 | 72 |
| **Patient No*.** | **Gender (1=Male 0=Female)** | **Age at Diagnosis** | **HGF score** | **HGF expression** | | **p-Fis1 score** | **p-Fis1 expression** | **p-Met score** | **p-Met expression** | **Over-all survival (OS) status (0=alive, 1=death)** | **OS time (Months)** |
| 1923 | 1 | 56 | 0 | Low | | 0.5 | Low | 6 | High | 0 | 67 |
| 1925 | 1 | 65 | 0 | Low | | 0.5 | Low | 4 | Low | 0 | 71 |
| 1927 | 1 | 39 | 1.5 | High | | 2.5 | High | 2 | Low | 1 | 3 |
| 1982 | 1 | 45 | 1 | Low | | 1 | Low | 4 | Low | 0 | 58 |
| 1953 | 1 | 56 | 1 | Low | | 1 | Low | NA |  | 0 | 69 |
| 2401 | 1 | 47 | 1 | Low | | 1 | Low | NA |  | 0 | 85 |

| **Table S3.** List of primary antibodies. | | |
| --- | --- | --- |
| **Antibody** | **Catalog Number** | **Company** |
| Met (D1C2) XP® Rabbit mAb | 8198 | Cell Signaling Technology |
| Phospho-Met (Tyr1234/1235) (D26) XP® Rabbit mAb | 3077 | Cell Signaling Technology |
| Phospho-Tyrosine (P-Tyr-1000) MultiMab™ Rabbit mAb mix | 8954 | Cell Signaling Technology |
| VDAC (D73D12) Rabbit mAb | 4661 | Cell Signaling Technology |
| HSP60 (D6F1) XP® Rabbit mAb | 12165 | Cell Signaling Technology |
| DYKDDDDK Tag Antibody | 2368 | Cell Signaling Technology |
| Met Antibody (C-28) | sc-161 | Santa Cruz Biotechnology |
| Anti-Fis1 Antibody (B-5) | sc-376447 | Santa Cruz Biotechnology |
| Tom20 Antibody (FL-145) | sc-11415 | Santa Cruz Biotechnology |
| Anti-MFF Antibody (B-2) | sc-398617 | Santa Cruz Biotechnology |
| Anti-Mfn1/Mitofusin 1 Antibody (D-10) | sc-166644 | Santa Cruz Biotechnology |
| Anti-Mfn2/Mitofusin 2 Antibody (F-5) | sc-515647 | Santa Cruz Biotechnology |
| Anti-OPA1 Antibody (D-9) | sc-393296 | Santa Cruz Biotechnology |
| Anti-SMCR7L Antibody (A-6) | sc-514135 | Santa Cruz Biotechnology |
| DRP1 (C-Terminal) Polyclonal Antibody | 12957-1-AP | Proteintech Group, Inc |
| GAPDH Monoclonal antibody | 60004-1-Ig | Proteintech Group, Inc |
| SMCR7/MID49 Polyclonal Antibody | 28718-1-AP | Proteintech Group, Inc |
| Monoclonal ANTI-FLAG® M2 antibody | F1804 | Sigma-Aldrich |
| Monoclonal Anti-α-Tubulin antibody | T5168 | Sigma-Aldrich |
| Anti-Actin antibody | A2066 | Sigma-Aldrich |
| Anti-Phosphotyrosine Antibody, clone 4G10® | 05-321 | Millipore |
| Anti-DNML1 antibody | H300010059-m01 | BD Biosciences |
| Anti-HGF antibody | ab83760 | Abcam |
| Antibody detecting phosphorylated Y38 Fis1 | custom-made | Zoonbio Biotechnology |
|  |  |  |

Movie S1.

N-SIM live cell imaging of mitochondrial localization of Met in HeLa cells transfected with mCherry–Met and EGFP–Mito.

Movie S2-3.

Live Cell Imaging of Huh7 cells showing mitochondrial fission events and mitochondrial morphology. Cells were treated with HGF (Supplementary movie S3) or not (Supplementary movie S2)

Movie S4-7.

Live Cell Imaging of Fis1^−/−^ Huh7 cells expressing empty vector (Supplementary movie S4), Fis1 WT (Supplementary movie S5), Fis1 Y38E mutant (Supplementary movie S6) or Fis1 Y38F mutant (Supplementary movie S7), showing mitochondrial fission events and mitochondrial morphology.

**References**

1 Ji, J. *et al.* XIAP Limits Autophagic Degradation of Sox2 and Is A Therapeutic Target in Nasopharyngeal Carcinoma Stem Cells. *Theranostics* **8**, 1494-1510, doi:10.7150/thno.21717 (2018).

2 Wieckowski, M. R., Giorgi, C., Lebiedzinska, M., Duszynski, J. & Pinton, P. Isolation of mitochondria-associated membranes and mitochondria from animal tissues and cells. *Nat Protoc* **4**, 1582-1590, doi:10.1038/nprot.2009.151 (2009).

3 Wettmarshausen, J. & Perocchi, F. Isolation of Functional Mitochondria from Cultured Cells and Mouse Tissues. *Methods Mol Biol* **1567**, 15-32, doi:10.1007/978-1-4939-6824-4_2 (2017).

4 Xiang, W. *et al.* Dietary fats suppress the peritoneal seeding of colorectal cancer cells through the TLR4/Cxcl10 axis in adipose tissue macrophages. *Signal Transduct Target Ther* **5**, 239, doi:10.1038/s41392-020-00327-z (2020).
